# Primary Thromboprophylaxis in Patients with Malignancies: Daily Practice Recommendations by the Hemostasis Working Party of the German Society of Hematology and Medical Oncology (DGHO), the Society of Thrombosis and Hemostasis Research (GTH), and the Austrian Society of Hematology and Oncology (ÖGHO)

**DOI:** 10.3390/cancers13122905

**Published:** 2021-06-10

**Authors:** Martin Kirschner, Nicole do Ó Hartmann, Stefani Parmentier, Christina Hart, Larissa Henze, Guido Bisping, Martin Griesshammer, Florian Langer, Ingrid Pabinger-Fasching, Axel Matzdorff, Hanno Riess, Steffen Koschmieder

**Affiliations:** 1 Department of Hematology, Oncology, Hemostaseology and Stem Cell Transplantation, Faculty of Medicine, RWTH Aachen University, 52074 Aachen, Germany; mkirschner@ukaachen.de (M.K.); nicole.doo@gmx.de (N.d.Ó.H.); 2 Center for Integrated Oncology Aachen Bonn Cologne Düsseldorf (CIO ABCD), 52074 Aachen, Germany; 3 Oncology and Hematology, Tumor Center, St. Claraspital, 4058 Basel, Switzerland; Stefani.Parmentier@claraspital.ch; 4 Department of Hematology and Oncology, Internal Medicine III, University Hospital Regensburg, 93053 Regensburg, Germany; christina.hart@ukr.de; 5 Department of Medicine, Clinic III—Hematology, Oncology, Palliative Medicine, Rostock University Medical Center, 18057 Rostock, Germany; larissa.henze@med.uni-rostock.de; 6 Department of Medicine I, Mathias Spital Rheine, 48431 Rheine, Germany; g.bisping@mathias-spital.de; 7 University Clinic for Hematology, Oncology, Haemostaseology and Palliative Care, Johannes Wesling Medical Center Minden, University of Bochum, 32429 Minden, Germany; Martin.Griesshammer@muehlenkreiskliniken.de; 8 II.Medical Clinic and Polyclinic, Center for Oncology, University Medical Center Hamburg-Eppendorf, 20246 Hamburg, Germany; langer@uke.de; 9 Clinical Division of Haematology and Haemostaseology, Department of Medicine I, Medical University of Vienna, 1090 Vienna, Austria; ingrid.pabinger@meduniwien.ac.at; 10 Department of Internal Medicine II, Asklepios Clinic Uckermark, 16303 Schwedt, Germany; a.matzdorff@asklepios.com; 11 Medical Department, Division of Oncology and Hematology, Campus Charité Mitte, Charité Universitätsmedizin Berlin, 13353 Berlin, Germany; hanno.riess@charite.de

**Keywords:** cancer, thrombosis, prophylactic anticoagulation, DOACs, Khorana risk score

## Abstract

**Simple Summary:**

Thromboembolic events occur in up to 20% of cancer patients during their course of disease. In addition, thromboembolism contributes relevantly to morbidity and mortality in this cohort. As a consequence, primary prophylaxis to prevent thromboembolic events is crucial. Due to enhanced bleeding risk in cancer patients, anticoagulation can be challenging in daily practice. Herein, we performed a systematic review regarding primary prophylaxis for thromboembolic events in cancer patients in order to aid clinicians in daily decision making. Besides general recommendations, specific subgroups were addressed, and recommendations are given at the end of each chapter. All topics were extensively reviewed and discussed among an expert panel including oncologists, hematologists, and hemostasis specialists, as members of the hemostasis working party of the German and Austrian Society of Hematology and Oncology as well as the German Society of Thrombosis and Hemostasis Research.

**Abstract:**

Patients with cancer, both hematologic and solid malignancies, are at increased risk for thrombosis and thromboembolism. In addition to general risk factors such as immobility and major surgery, shared by non-cancer patients, cancer patients are exposed to specific thrombotic risk factors. These include, among other factors, cancer-induced hypercoagulation, and chemotherapy-mediated endothelial dysfunction as well as tumor-cell-derived microparticles. After an episode of thrombosis in a cancer patient, secondary thromboprophylaxis to prevent recurrent thromboembolism has long been established and is typically continued as long as the cancer is active or actively treated. On the other hand, primary prophylaxis, even though firmly established in hospitalized cancer patients, has only recently been studied in ambulatory patients. This recent change is mostly due to the emergence of direct oral anticoagulants (DOACs). DOACs have a shorter half-life than vitamin K antagonists (VKA), and they overcome the need for parenteral application, the latter of which is associated with low-molecular-weight heparins (LMWH) and can be difficult for the patient to endure in the long term. Here, first, we discuss the clinical trials of primary thromboprophylaxis in the population of cancer patients in general, including the use of VKA, LMWH, and DOACs, and the potential drug interactions with pre-existing medications that need to be taken into account. Second, we focus on special situations in cancer patients where primary prophylactic anticoagulation should be considered, including myeloma, major surgery, indwelling catheters, or immobilization, concomitant diseases such as renal insufficiency, liver disease, or thrombophilia, as well as situations with a high bleeding risk, particularly thrombocytopenia, and specific drugs that may require primary thromboprophylaxis. We provide a novel algorithm intended to aid specialists but also family practitioners and nurses who care for cancer patients in the decision process of primary thromboprophylaxis in the individual patient.

## 1. Introduction

With physicians being aware of the increased risk of thromboembolic complications in cancer patients and the high impact of these events on quality of life, morbidity, and mortality as well as the connected economic burden, studies on the role of primary pharmacological prophylaxis in different populations of cancer patients started more than 20 years ago, and some more recent studies are ongoing.

A venous thrombotic/thromboembolic event (VTE) is a frequent and serious complication of cancer. VTEs in cancer patients not only cause significant morbidity and mortality [1], they are also a ‘signum malum’, indicating an adverse prognosis of the cancer itself [2,3]. This negative prognostic effect has been shown for all common cancer types, e.g., gastrointestinal cancer [4], lung cancer [5,6], breast cancer [7] prostate cancer [8], and hematological malignancies [9]. This adverse prognosis has been attributed to several factors [10,11]: first, VTEs may directly lead to death. It has been shown that, while most cancer patients die from tumor progression, thromboembolism (both venous and arterial events) ranks second as a cause of death [1]. The tumor itself also activates coagulation. Biologically active tumors release thrombogenic material, and VTE is an indicator of cancer cell proliferation [10,12,13]. Moreover, patients who have a progressive tumor are more likely to undergo surgery, systemic anti-cancer therapy, or other medical interventions that increase the risk of VTE [11,14,15,16]. VTE risk is particularly high during the first months of cancer treatment [7,10]. Finally, besides direct adverse effects, VTEs may cause delays or even discontinuation of cancer treatment with a negative impact on prognosis. Despite these well-recognized interactions, clinical trials have failed to demonstrate a survival benefit for primary thromboprophylaxis in cancer patients generally [17,18,19,20]. Moreover, while several randomized clinical trials have shown that thromboprophylaxis reduced VTE-related morbidity in cancer patients [17,19,20,21,22,23,24,25,26], albeit mostly at an increased bleeding risk, other studies have failed to show significantly reduced VTEs [16,18]. Here, we review current evidence for primary thromboprophylaxis in cancer patients and provide recommendations on its use for daily practice scenarios. Besides recommendations after each paragraph, all recommendations are summarized in a comprehensive table in the supplement (Supplementary Materials Table S1).

## 2. Material and Methods

By review of publications (up to 15 August 2020 in English or German) in Pubmed (http://www.ncbi.nlm.nih.gov/pubmed, accessed 15 August 2020) using the following keywords: “primary thromboprophylaxis AND cancer”. 259 publications were identified. Other publications were subsequently selected based on the specific scenarios described in this review, which had been defined by the group of co-authors.

## 3. Results and Recommendations: General Recommendations for Primary Thromboprophylaxis in Patients with Malignancies

### 3.1. Studies in Unselected Cancer Patient Cohorts

#### 3.1.1. VTE Prevention in Patients with Cancer Hospitalized Due to Medical Reasons

**Figure 1:**
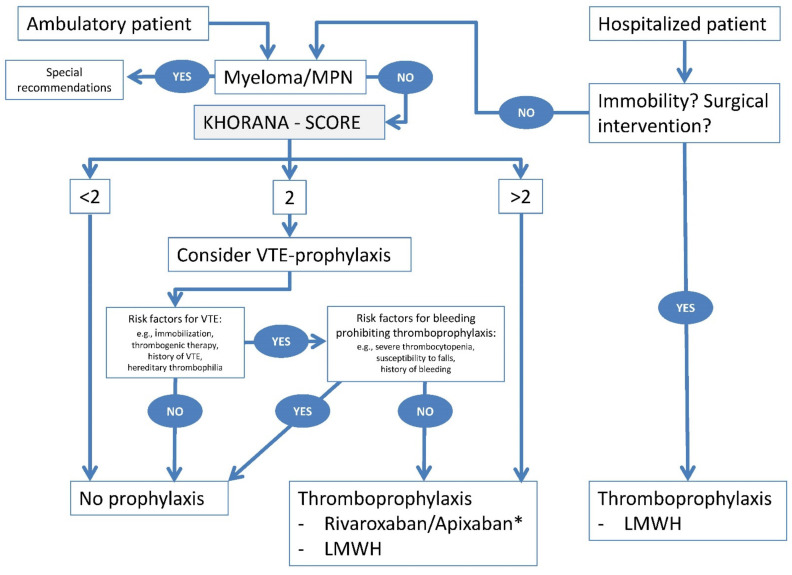
Algorithm for individual decisions for primary thromboprophylaxis in cancer patients. Of note, each indication of thromboprophylaxis has to be carefully balanced with regard to the individual bleeding risk. A hospitalized patient should be assessed for immobility and/or a planned surgical intervention. In both of these situations, thromboprophylaxis is indicated in all patients (unless there is a contraindication) with low-molecular-weight heparin (LMWH). In ambulatory, non-hospitalized patients or hospitalized patients who are not immobile and are not undergoing surgery, and who do not carry a diagnosis of multiple myeloma or myeloproliferative neoplasm (MPN) (see special Section 4.6 and Section 4.8 for these patients), the Khorana score should be calculated. Such patients with a Khorana score of <2 are typically not candidates for thromboprophylaxis. Patients with a Khorana score of >2 should receive thromboprophylaxis, using either rivaroxaban or apixaban, or an LMWH. Finally, we recommend that, in patients with a Khorana score of 2, primary thromboprophylaxis should be considered based on the presence or absence of individual additional risk factors for venous thrombotic/thromboembolic events (VTE) in the patient. If the patient with a Khorana score of 2 has no additional risk factors (such as immobilization at home, thrombogenic therapy, history of VTE, and/or hereditary thrombophilia), this patient is not considered a candidate for primary thromboprophylaxis. However, in the presence of one or more of these VTE risk factors, the risk factors for bleeding which prohibit thromboprophylaxis should be addressed (e.g., severe thrombocytopenia, susceptibility to falls, and/or history of bleeding). If such a risk factor is present, this argues against thromboprophylaxis; if not, we recommend thromboprophylaxis in such a patient. * DOAK not approved for primary prophylaxis in ambulatory cancer patients.

Large studies on prophylactic anticoagulation, as compared to placebo, in patients with acute medical illness have demonstrated a risk reduction for VTE without a significant increase in bleeding events [27,28,29] (Table 1), with a favorable safety profile of low-molecular-weight heparins (LMWHs) compared to unfractionated heparin (UFH) [30]. In these studies, only 5 to 15% of patients had cancer. LMWH and UFH showed similar efficacy and safety in a subgroup of hospitalized patients with cancer [31]. According to a systematic meta-analysis of more than 300 hospitalized cancer patients, out of a subgroup of almost 5000 patients from three randomized trials, prophylactic anticoagulation with LMWH or fondaparinux, as compared to placebo, was unable to provide a benefit in VTE reduction [16]. No cancer-specific in-patient thromboprophylaxis trials have been conducted. Nevertheless, most guidelines [32,33,34,35] support the use of pharmacological prevention in cancer patients admitted to the hospital with an acute medical illness. For mobile cancer patients admitted to the hospital for non-surgical diagnostic purposes or for complex anti-cancer therapies, the indication for thromboprophylaxis should follow the recommendations for outpatients, since the Khorana score (KS, Table 2) has shown validity in predicting VTE development in hospitalized patients as well [36] (see Figure 1 and below).

On the other hand, non-hospitalized but “immobilized” cancer patients should be considered for thromboprophylaxis similarly to hospitalized patients (Figure 1). The term “immobilized patients” is used here to denote patients who are bedbound, unable to walk unaided, or likely to spend a substantial proportion of the day in bed or in a chair. Immobilization is a generally recognized risk factor for VTE of its own.

#### 3.1.2. VTE Prevention in Ambulatory Patients with Cancer Receiving Anti-Cancer Therapy

The risk of symptomatic VTE shows wide variability in ambulatory cancer patients based on different cancer types, cancer stages, anti-cancer, and supportive treatments, as well as cancer-independent patient characteristics. A major obstacle is the fact that, due to the different incidences of specific cancers, those with a relatively low VTE risk such as breast and prostate cancer nevertheless are responsible for the major burden of cancer-associated VTE because of the high prevalence of these entities [40]. Consequently, large studies with unselected cancer patients undergoing anti-cancer treatment showed significant reductions in VTE by prophylactic anticoagulation with LMWH, but the event rates and the absolute differences were low [23,37] (Table 1). Two recent Cochrane Database analyses confirm the significant—close to 50%—reduction of symptomatic VTE by LMWH or fondaparinux without significant increases in major bleeding, but absolute VTE rates were low [41,42]. Because of the low incidence of VTE in the outpatient setting, primary pharmacological prevention is not recommended routinely to all cancer outpatients receiving systemic anti-cancer therapy [33,34,35,36].

Recommendations:

- Based on recommendations for hospitalized patients due to medical illness in general, we recommend prophylactic anticoagulation for cancer patients in an in-patient setting, except for fit/mobile cancer patients admitted due to non-surgical procedures. In these cases, recommendations for outpatients should be followed (see below).
- Due to the wide variability of VTE risk in ambulatory cancer patients, we do not recommend primary pharmacological prophylaxis in general. This topic will be discussed in the following paragraphs. Refer also to Figure 1.

### 3.2. Studies in Selected Cancer Patient Cohorts

#### 3.2.1. Low-Molecular-Weight Heparins (LMWHs)

Prospective randomized trials in a few selected cancers, clinically identified as having a high risk for VTE, showed better risk-benefit ratios. In patients with advanced pancreatic cancer undergoing systemic chemotherapy, LMWH administered at half—(1 mg/kg enoxaparin once daily [QD]) or full-therapeutic (initially 200 anti-Xa units/kg, after 4 weeks 150 anti-Xa units/kg of dalteparin QD) dosage significantly reduced the VTE rate by more than 80% without an increase in bleeding complications [22] (Table 1). Interestingly, the rate of arterial thromboembolic events, which is also increased in cancer patients compared to non-cancer patients, was investigated in one of the studies and numerically decreased [26].

A recent placebo-controlled trial in 390 patients with systemically treated small-cell lung cancer (SCLC) assessed the effect of a half-therapeutic dose of LMWH (1 mg/kg enoxaparin QD during chemotherapy treatment) on mortality. There was no benefit with regard to progression-free or overall survival, but the VTE rate was significantly reduced [38] (Table 1). In another randomized open-label study in 549 NSCLC patients, LMWH (100 anti-Xa units/kg tinzaparin QD for 12 weeks) after tumor resection failed to demonstrate a beneficial effect on 5-year survival as well as on VTE rate [43].

Due to the specific aspect of anticoagulation in myeloma patients, this topic will be discussed in a separate section.

Prospective randomized clinical studies based on VTE risk assessment tools aimed to investigate the role of anticoagulant drugs in the primary prevention of VTE in an *a priori* defined high VTE risk population with different underlying cancers. All prospective randomized trials completed today have used the Khorana Score (KS) to categorize the individual VTE risk. In fact, a subgroup analysis of the PROTECHT study [37] confirmed the greatest benefit (number needed to treat [NNT] 15) of LMWH (3800 anti-Xa units nadroparin QD) for high-risk patients, i.e., those with a Khorana Score ≥ 3 [44].

A first study investigated LMWH (5000 anti-Xa units dalteparin QD) for 12 weeks or observation in patients with KS ≥ 3. After excluding 8.5% of patients with a pre-existing asymptomatic VTE by ultrasound screening, 98 cancer patients without VTE were randomized. The 12-week rate of symptomatic and asymptomatic events was numerically decreased from 21 to 12% by LMWH, but due to premature termination, because of slow patient accrual, the study was underpowered to show statistical significance. In contrast to major bleeding, the rate of clinically relevant non-major bleeding (CRNMB) was significantly higher in the dalteparin group [18].

#### 3.2.2. Direct Oral Factor Xa Inhibitors (DOACs)

The role of Direct Oral AntiCoagulants (DOACs) has also recently been assessed for two factor Xa inhibitors (apixaban and rivaroxaban) in cancer patients with KS ≥ 2 (this threshold differs from the Khorana high-risk definition and includes some intermediate-risk patients since the overall VTE risk of this group is almost 10% in 6 months [45]). The AVERT study [17] compared 2.5 mg of apixaban twice daily (BID) to a placebo for 180 days in 573 cancer patients receiving chemotherapy. Patients in the apixaban arm had significantly less symptomatic and incidental VTE (4.2 vs. 10.2%; number needed to harm [NNH] 17) and a significantly increased risk of major bleeding (3.5 vs. 1.8%; NNH 59). As patients prematurely stopped drug intake (median treatment duration: 156 d), the "on-treatment" analysis showed an improved efficacy (VTE: 1.0 vs. 7.3%; NNT 16) and the risk of major bleeds lost significance (2.1 vs. 1.1%; NNH 100).

The CASSINI study [39] compared 10 mg of rivaroxaban QD to a placebo for 180 days in cancer patients initiating chemotherapy. Prior to randomization, 4.5% of ultrasound-screened KS ≥ 2 patients were excluded from the study because of asymptomatic deep vein thrombosis (DVT). 841 patients were randomized. In the intention-to-treat analysis, the composite endpoint of symptomatic and asymptomatic DVT, pulmonary embolism, and VTE-related death was non-significantly decreased (6.0 vs. 8.8%; NNT 36). Major bleeding events were numerically doubled (2.0 vs. 1.0%; NNH 100). Furthermore, arterial thromboembolic events were collected in the CASSINI patients, confirming a numerical reduction (1.0 vs. 1.7%, NNT 143) by primary thromboprophylaxis. "On-treatment" analysis with a mean intervention period of 4.3 months resulted in a significant reduction in the primary endpoint (2.6 vs. 6.4%; NNT 26) without a change in the rate of major bleeding.

Available subgroup analyses of the CASSINI trial confirm the significant efficacy (VTE 3.7 vs. 10.1%, NNT 16, on treatment analysis) and safety (major bleeding 1.5 vs. 2.3%) of pharmacological thromboprophylaxis in the stratified and pre-specified subgroup of 273 patients with pancreatic cancer. In this cancer, 6.6% of patients were *a priori* excluded from the study due to asymptomatic DVT detected by screening ultrasound.

In a pre-specified on-treatment (defined as the period from the first dose of study medication to 2 days after last application) subgroup analysis of the CASSINI trial, those 68.5% of patients with KS = 2 showed a similar—formally significant—reduction in the primary endpoint (2.1 vs. 6.4%, NNT 23) as the whole population. In the AVERT trial, the post-hoc analysis demonstrated a significant reduction in VTE in the KS 2 cohort of 376 patients [46] (3.2 vs. 8.4%, NNT 19). Keeping the different designs of the two studies in mind, these data demonstrate a clinically relevant high VTE risk in patients with KS 2, which is effectively reduced by the factor Xa inhibitors investigated. Of note, no trials exist for edoxaban and dabigatran in this setting.

To investigate whether another score, different from the Khorana score, could improve risk stratification, the investigators of the AVERT trial sought to identify and validate a more efficient venous thromboembolism (VTE) risk threshold for thromboprophylaxis [47]. This score is based on the tumor entity and a biomarker (D-Dimer) only [48]. The score can be assessed using a published online calculator: catscore: Cancer-associated VTE—clinical prediction model (meduniwien.ac.at). In a modified intention-to-treat analysis, this score was assessed with regard to efficacy (VTE) and safety (major and overall bleeding) in a post-hoc analysis of the (a) complete AVERT cohort and (b) ≥8% and < 8% 6-month VTE risk thresholds. Numbers needed to treat (NNT) to prevent one VTE in patients on apixaban compared to placebo were improved by this score from 17 to 6 by the use of the catscore in patients with a predicted 6-month VTE risk ≥8%. Rates of VTE events were 8.4% in patients on apixaban and 26.3% on those with placebo (aHR 0.33 [0.14–0.81], *p* < 0.05). Individuals with a VTE risk <8% derived no benefit from apixaban thromboprophylaxis (aHR 0.89 [0.30–2.65), *p* = 0.84). Bleeding was increased in all of the groups on apixaban.

It is worthwhile to recall some facts before summarizing the available evidence and to balance the benefits and risks of pharmacological thromboprophylaxis in ambulatory cancer patients undergoing systemic anticancer treatment beyond tumors with good evidence such as pancreatic cancer or multiple myeloma:

(i) Mortality due to VTE complications in cancer patients is much higher than mortality due to bleeding complications [1].
(ii) The International Society on Thrombosis and Haemostasis (ISTH) definition for major bleeding [49] applied in clinical trials has never been validated in cancer patients undergoing therapy. This is important, as the two main criteria to categorize a major bleeding event according to ISTH, namely a 2 g/dL drop of hemoglobin and transfusion requirement, are well-known events in non-bleeding cancer patients undergoing systemic cancer treatment. It should thus be realized that bleeding events in this patient population are up-graded in most of the events from “minor” to “major”.
(iii) Despite the KS being validated for many cancer patients, this scoring system is not discriminative in all cancers [50], and it does not include well-known thrombogenic risk factors such as the history of VTE, highly elevated D-dimer levels, age, degree of mobilization, presence of thrombocytopenia, or thrombogenic or pro-hemorrhagic anti-cancer therapies. One other prediction model has been demonstrated in a post-hoc analysis of an interventional study to allow an improved risk prediction and decreased NNT compared to the Khorana score (Table 2).

**Table 2:** KHORANA Score [44].

Patients’ Characteristics	Score
Site of cancer:	
– Very high risk (pancreas or stomach) – High risk (lung, lymphoma, gynecological, bladder, or testicular)	+2 +1
Prechemotherapy platelet count 350 × 10^9^/L or higher	+1
Hemoglobin level less than 100 g/L or use of red cell growth factors	+1
Prechemotherapy leucocyte count more than 11 × 10^9^/L	+1
BMI 35 kg/m^2^ or more	+1

Classification: ≥3 points: high risk, 1–2 points: intermediate risk, 0 points: low risk.

Recommendations:

- The available evidence is in favor of primary pharmacological prevention (LMWH either prophylactic or half-therapeutic dose in certain cancer types such as pancreatic cancer, or apixaban 2 × 2.5 mg or rivaroxaban 1 × 10 mg) in ambulatory cancer patients undergoing systemic anti-cancer therapy with a Khorana score (KS) of > 2 or, although less evidence-based, those identified with other risk scores or with other high-risk factors for VTE. However, given the limited available data, the panel agreed that any such recommendation must be made on an individual basis, and it should take into account the patient´s preference.

### 3.3. Drug Interactions in Patients with Malignancies Receiving Prophylactic Anticoagulation

All oral anticoagulants exhibit interactions with other drugs. Interactions of vitamin K antagonists (VKAs) may be controlled through dose adjustment upon INR measurements. DOACs have fewer drug interactions than VKAs, but no standardized response monitoring has been established. Therefore, for adequate management, it is crucial to understand the mechanisms of potential interactions. DOACs are mainly metabolized through P-glycoprotein (P-gp) and cytochrome P (CYP) metabolic pathways. Therefore, drug interactions have to be considered with drugs that are also metabolized via these pathways. In addition to medications for cardiovascular or other comorbidities, some chemotherapeutic agents such as adriamycin and vincristine can reduce the plasma concentration of DOACs through P-gp and CYP3A4 induction. Tyrosine kinase inhibitors, endocrine therapies, and others can influence the plasma levels of DOACs through both of the above-mentioned pathways. For detailed information, we recommend using CYP interaction websites [51,52,53].

Apart from drug interactions, the bioavailability of DOACs also needs to be considered. Modifications in gastrointestinal transit time or acidity can influence the bioavailability of DOACs. Solubility of rivaroxaban, edoxaban, and dabigatran is pH-dependent. Therefore, these DOACs require gastric acidity for their absorption. These drugs are mainly absorbed in the distal stomach and proximal small bowel; therefore, bioavailability is likely to decrease after gastrectomy or gastric bypass surgery [52,54]. Apixaban is absorbed in a pH-independent manner, and up to one-half of the drug is absorbed in the distal small bowel and ascending colon. That is why apixaban should be avoided in patients who have undergone small bowel resection or colectomy [55]. Rivaroxaban needs to be taken with a meal, while apixaban and dabigatran can be taken independently from meals. Therefore, decreased efficacy due to malabsorption and malassimilation or increased bleeding risk due to decreased metabolism is possible for DOACs. Currently, there are no standardized guidelines on how to apply DOAC drug monitoring or guidelines for risk assessment and dose modifications for DOACs. Therefore, in such situations, the use of LMWH may be a safer and more predictable option [52].

Recommendations:

- If prophylactic anticoagulation is indicated, the choice of optimal oral drug should be considered on an individual basis taking into account drug interactions and the bioavailability (e.g., patients after abdominal surgery). In uncertain cases, LMWH should be preferred to DOACs.

## 4. Specific situations of Prophylactic Anticoagulation in Patients with Malignancies

### 4.1. Peri-Interventional Thromboprophylaxis in Patients with Cancer Undergoing Elective Invasive Procedures

Postoperative VTE occurs more frequently among cancer compared to non-cancer patients [56], which is reflected by two points for the presence of malignancy in the Caprini score (surgical scoring system containing several factors to assess the risk of postoperative thromboembolic events) [57].

Several clinical trials have confirmed the efficacy of low-dose UFH, LMWH, and fondaparinux (FPX) in preventing DVT and PE in patients undergoing major surgery, without significantly increasing bleeding complications. Compared to UFH, LMWHs have proven beneficial through a more predictable anticoagulant response, a longer plasma half-life, better bioavailability when administered subcutaneously, a lower risk of heparin-induced thrombocytopenia, and the convenience of once-daily dosing. A meta-analysis incorporating 16 randomized trials with 12,890 patients with cancer showed no difference in safety or efficacy comparing prophylactic LMWH to prophylactic UFH in the postoperative setting [58,59,60].

Moreover, extending the duration of prophylaxis after surgery was beneficial in cancer patients, as shown in the ENOXACAN II and CANBESURE studies. In the ENOXACAN II study [61], a significantly lower rate of VTE in cancer patients treated with prophylactic doses of LMWH for at least 1 month after surgery was observed than in those treated for only 1 week (13.8 vs. 5.5%). The CANBESURE study compared one week of prophylactic bemiparin with 4 weeks of prophylaxis in cancer patients undergoing major abdominal or pelvic surgery [24]. Although the study failed to show a significant reduction in the composite endpoint of VTE plus all-cause mortality with extended prophylaxis (10.1 vs. 13.3%, respectively; *p* = 0.26), there was a significant reduction in major thrombotic events with extended prophylaxis (0.8 vs. 4.6%, *p* = 0.01) [24]. Other studies and meta-analyses have demonstrated a significant reduction in proximal VTE using extended prophylaxis [62,63,64,65,66]. The results, therefore, suggest that the major benefit of such prophylaxis may be the prevention of relevant thrombotic complications [62,63,64,65,66]. A recent meta-analysis including 39 studies comparing perioperative pharmacological thromboprophylaxis in cancer patients undergoing surgery with no pharmacological prophylaxis (including mechanical prophylaxis or no prophylaxis) demonstrated a 50% reduction in the rate of DVT with pharmacological prophylaxis (DVT incidence 0.5 vs. 1.2%, RR 0.51, 95% CI 0.27–0.94; *p* = 0.03), with an expected increase in the risk of bleeding, but without a difference in mortality or pulmonary embolism (PE) [67]. Unfortunately, in this meta-analysis, major and minor bleeding events were not differentiated, which makes an “acceptable increase in the risk of bleeding” questionable. In addition, a systematic review and meta-analysis of seven randomized studies (encompassing 4807 patients) showed that extended thromboprophylaxis (2–6 weeks) after abdominopelvic cancer surgery significantly reduced the risk of all VTE and proximal DVT by approximately 50%, when compared with the conventional duration of thromboprophylaxis (<2 weeks) [68]. No difference was found in the incidence of symptomatic PE, major bleeding events, or 3-month all-cause mortality [68].

According to these results, thromboprophylaxis with LMWH, UFH, or fondaparinux is recommended for most cancer patients undergoing surgery with a duration of more than 30 min and without contraindications (bleeding, prolonged thrombocytopenia, and platelets lower than 30 G/L) for the duration of their hospitalization (in general for 6–10 days). For patients undergoing abdominopelvic cancer surgery, extended prophylaxis (4–5 weeks) is recommended [69,70,71]. For surgical patients with contraindications for anticoagulation, including cancer patients, mechanical prophylaxis (compression stockings or intermittent pneumatic compression in high-risk patients) is an effective alternative as long as no contraindications exist (e.g., decompensated heart failure, thrombophlebitis, thrombosis or suspected thrombosis, acute limb infections, severe uncontrolled hypertension or compartment syndrome) [35,71,72].

Recommendations:

- Prophylactic anticoagulation in cancer patients undergoing major abdominal or pelvic surgery should be extended to 4–5 weeks after surgery.
- Thromboprophylaxis should be performed with LMWH, UFH (second choice), or fondaparinux. The exact peri-operative timing of these drugs is described in the respective Summary of Product Characteristics (SmPC).
- Mechanical prophylaxis (compression stockings or intermittent pneumatic compression) may be performed in patients with contraindications for anticoagulation.

### 4.2. Thromboprophylaxis in Patients with Cancer and Renal Insufficiency

Renal insufficiency is common in patients with malignancies. A large multicenter retrospective study, the Renal Insufficiency and Anticancer Medications (IRMA) study [73] analyzed renal function parameters in 4684 patients. The percentage of patients with stage 1 (GFR ≥ 90 mL/min), stage 2 (GFR 60–89 mL/min), stage 3 (GFR 30–59 mL/min), and stage 4/5 (GFR < 30 mL/min) renal insufficiency, as calculated by the MDRD formula, was 37.7%, 40.9%, 11.1%, and 0.92% of patients, respectively. Renal dysfunction itself is an important risk factor for venous thromboembolism [74], but, at the same time, the risk of bleeding in cancer patients receiving anticoagulation for VTE is enhanced by renal dysfunction [75]. On the other hand, since renal function affects the metabolism and elimination of most drugs, renal insufficiency has to be taken into account when treating patients with prophylactic anticoagulation. Of note, the bleeding risk between LMWH for thromboprophylaxis was not different from that with UFH in non-cancer patients with end-stage renal disease [76,77]. Table 3 lists the recommended dose adjustments for the most common anticoagulants in patients with altered renal function. Since DOACs have not been approved for thromboprophylaxis in cancer patients, dosing and dose adjustments for renal dysfunction are based on recommendations for post-surgery prophylaxis. It has to be noted that recommended dose adjustments for renal dysfunction are not based on firm clinical evidence but rather on risk assessment and retrospective drug level correlations. Furthermore, these dose-adjustment recommendations are subject to frequent updates, therefore, we recommend consultation of the prescribing information to determine the final necessary dose.

Recommendations:

- In patients with renal dysfunction, the risk of bleeding during thromboprophylaxis is increased. However, no firm clinical evidence exists on how to adjust the dose of thromboprophylactic agents. Therefore, we recommend a very thorough assessment of the necessity for and the dose of pharmacologic thromboprophylaxis in patients with reduced eGFR, particularly those with an eGFR below 30 mL/min. The risk of bleeding has to be weighed against the risk of thromboembolism.
- Anti-Xa plasma level monitoring during LMWH thromboprophylaxis is helpful in patients with stage 4 to 5 chronic kidney disease, with optimal levels (3–4 h after injection) being 0.1 to 0.3 IU/mL for prophylactic dosing [78]. However, clinical judgment currently remains the main factor in deciding on the dose of these agents.

### 4.3. Thromboprophylaxis in Patients with Cancer and Liver Disease

The burden of liver disease in the general population is high [79]. Thus, liver disease is also common in cancer patients and may be caused by preexisting medical conditions (e.g., liver cirrhosis), liver metastases, toxicity from cancer immuno- or chemotherapy [80], and/or from infections during cancer treatment. At the same time, liver cirrhosis is currently considered a prothrombotic condition due to increased factor VIII and von Willebrand factor and decreased antithrombin III and protein C levels [81,82], which adds to the already increased VTE risk in cancer patients. In addition, portal vein thrombosis is common in liver disease and almost ⅔ are associated with hepatobiliary malignancy, and 3% with myeloproliferative diseases [83]. Thromboprophylaxis in cancer patients with liver disease is therefore not an uncommon clinical issue. However, the major concern about anticoagulants has always been that they increase bleeding risk, which is also high with liver disease [84].

There is a scarcity of data on the safety and efficacy of anticoagulants in patients with liver disease. This is because patients with clinically relevant liver disease have usually been excluded from anticoagulant trials. In the prescribing information of anticoagulants, one often finds that they should be avoided in patients with "severe" liver disease or in liver disease with “coagulopathy”. One has to keep in mind that there is no consensus on what defines severe liver disease and which laboratory test and which threshold, e.g., AST > 2 times the upper limit of normal, indicates an increased bleeding risk. The often-applied Child-Pugh criteria have been developed to predict survival in liver cirrhosis, but not liver cancer or bleeding risk.

For a long time, the efficacy of LMWH was thought to be unpredictable in liver disease, owing to the fact that LMWH requires antithrombin for its function and that antithrombin levels are often lower than normal [84]. However, there is at least one randomized study that showed that fixed prophylactic dose LMWH is effective and safe in preventing portal vein thrombosis in cirrhotic patients [85]. There is only limited data on the safety and efficacy of DOACs in patients with liver disease because this subgroup has usually been excluded from recent DOAC-trials. When using DOACs in cancer patients with liver disease, one has to consider hepatic metabolism. Rivaroxaban is processed by both the liver and kidneys, with about two-thirds of the drug metabolized by the liver. Rivaroxaban seems to be affected more by liver disease than apixaban. The prescribing information recommends avoiding rivaroxaban in patients with Child B liver cirrhosis due to an increase in drug exposure. Recently, the European Heart Rhythm Association published a recommendation for atrial fibrillation patients with liver disease to prefer apixaban [86]. We suggest that this can be applied in a similar fashion to cancer patients with liver disease.

It has been reported that rivaroxaban may itself induce liver injury, at least more commonly than other DOACs, warfarin, and LMWH [87,88], and for this reason, caution should be exerted using rivaroxaban in thromboprophylaxis in patients with cancer and liver disease.

Recommendations:

- There are no evidence-based recommendations on prophylaxis of cancer-associated VTE in patients with severe liver disease. We recommend individualized treatment regimens after shared decision-making with patients.
- Prophylactic dose anticoagulation may usually be offered since the bleeding risk is low. Careful clinical and laboratory monitoring is suggested, especially in patients with prolonged prothrombin time.
- In patients with liver disease and a history of severe bleeding, e.g., from esophageal varices, it might be prudent not to use pharmacological thromboprophylaxis.
- Make sure the patient has been informed about clinically relevant bleeding symptoms and about signs of thromboembolism. He/She should know whom to contact when developing such symptoms and where to go after office hours or on the weekend.

### 4.4. Thromboprophylaxis in Patients with Cancer and Elevated Bleeding Risk

Elevated bleeding risk is a frequent problem in the clinical management of cancer patients. Three main underlying factors have to be considered in this situation: (1) tumor-site specific, (2) individual and/or situational, and (3) drug-specific risk factors for specific bleeding risk [89,90,91,92,93].

(i) Tumor-site specific bleeding risk: patients with glioblastoma/CNS tumors carry a high risk for the development of VTE in the first six-month period from diagnosis [94,95]. Prophylactic application of LMWH non-significantly decreases the VTE incidence but, in contrast, is associated with a higher risk of intracranial bleeding [96,97]. Nevertheless, prophylactic anticoagulation is generally recommended in this cohort [98]. In addition, individually risk-adapted algorithms addressing the safety of primary VTE prophylaxis (i.e., prevention of VTE on the one and clinically relevant major bleedings (CRMB) on the other hand) should be applied for lymphomas (depending on the stage and sites of lymphoma), lung tumors (with respect to the involvement of arterial vessels and/or mediastinal, pleural or pericardial manifestations) and tumors of the genitourinary tract in general.
(ii) Individual/situational bleeding risk factors: renal impairment, liver dysfunction, and low platelet counts are addressed in separate sections. Moreover, platelet dysfunction, preexisting bleeding disorders (i.e., coagulopathy or (acquired) von Willebrand Syndrome), history of severe bleeding episodes, as well as poor functional status, and age of > 75 years may contribute to an increased bleeding risk. On the other hand, perioperative and periprocedural settings can implicate a higher risk of local (mucosal, intracranial, or intraspinal) or systemic bleeding.
(iii) Drug-specific risk factors for bleeding: tumor-specific treatment (i.e., BTK inhibitor—ibrutinib or inhibitors of VEGFR-associated tyrosine kinases i.e., axitinib, cabozantinib, lenvatinib, nintedanib, pazopanib, regorafenib, sorafenib, sunitinib, tivozanib or vandetanib) and/or concomitant pain medication (e.g., NSAIDs) may contribute to an increased risk of bleeding. These drugs have to be handled carefully with frequent monitoring of clinical aspects indicating increased individual bleeding risk.

Recommendations:

- Tumor-site specific risk: an individual assessment of bleeding risk has to be carried out, especially in patients with glioblastomas, lymphomas, gastrointestinal cancers, lung cancers, and tumors of the genitourinary tract.
- Individual/situational risk: laboratory tests reflecting organ function and platelet count as well as age, bleeding history have to be evaluated carefully. In the context of interventional procedures, management and scoring of bleeding risk are recommended for individual adaptation of thromboprophylaxis.
- Drug-specific risk factors: bleeding risk has to be assessed and monitored according to the anti-cancer medication and other co-medication.

### 4.5. Thromboprophylaxis in Patients with Cancer and Thrombocytopenia

Thrombocytopenia is common in patients with malignancies. Up to 24% of patients with solid tumors develop thrombocytopenia due to chemotherapy [99]. In hematologic malignancies, thrombocytopenia is even more common and may also be caused by the malignant disease itself [100]. In general, low platelet counts increase the risk for bleeding, especially platelet counts below 10 G/L [101]. Conversely, low platelet counts are not protective against thromboembolism [100,102]. Consequently, thromboprophylaxis may be beneficial in thrombocytopenic patients depending on their individual risk for thrombosis, the severity of thrombocytopenia, and other risk factors for bleeding. No dedicated trials have evaluated bleeding risk relative to platelet count in any population of patients requiring prophylactic anticoagulation [103]. Retrospective and prospective observational analyses show that prophylaxis with LMWH is possible in patients with very low platelet counts (1 to 18 G/L) [102,104]. Nevertheless, the AVERT and CASSINI trials investigating apixaban and rivaroxaban respectively as thromboprophylaxis in ambulatory patients with cancer excluded patients with platelet counts below 50 G/L and interrupted study medication if platelets fell below 50 G/L [17] or 25 G/L [39] during study participation.

A recent survey in France described the diverse opinions of physicians towards prophylactic anticoagulation in patients with thrombocytopenia [105]. Of 98 physicians (among them 71 hematologists and 5 oncologists), 5% considered a platelet count of <80 G/L as relevant to refrain from thromboprophylaxis, whereas most agreed with a threshold of <50 G/L (26%) or <30 G/L (48%) not to administer thromboprophylaxis. 6% would apply thromboprophylaxis in patients with platelet counts as low as 10 G/L, and 12% of physicians irrespective of thrombocytopenia.

Current guidelines and recommendations acknowledge the low level of evidence: European [34,99,106,107]., American [108], Canadian [103], and international guidelines [109,110,111] and a recent critical review [112], all dating from 2013 to 2019, on thromboprophylaxis in hospitalized cancer patients or other medical patients with thrombocytopenia. Most guidelines consider thromboprophylaxis as not contraindicated if platelet counts are above 50 G/L [110]. Nevertheless, no conclusive recommendation is given for platelet counts below 50 G/L, and most guidelines recommend a decision on a case-by-case basis in these patients. The international guidance of the ISTH [109] and the German guideline of DGHO (German Society of Hematology and Medical Oncology) are precise about stopping prophylactic anticoagulation in patients with platelet counts below 25 G/L or 30 G/L, respectively.

Recommendations:

In thrombocytopenic patients:

- we recommend against thromboprophylaxis in cancer patients, if platelet counts are <25 G/L.
- pharmacologic thromboprophylaxis with LMWH may be considered if platelet counts are between 25 and 50 G/L.
- we recommend against mechanical prophylaxis (pneumatic intermittent compression or compression stockings) in patients with platelet counts <50 G/L.

### 4.6. Thromboprophylaxis in Patients with Cancer and Thrombophilia

Patients with malignancies and co-existing thrombophilia require special attention and potentially alternative prophylactic anticoagulation. Thrombophilia is defined as an increased risk of thromboembolism due to specific hereditary or acquired causes. The most prevalent types and causes are shown in Table 4. Prospective data on prophylactic anticoagulation are lacking for most of these patients, and no explicit approval has been granted for anticoagulants for thromboembolism prophylaxis in patients with malignancies and co-existing thrombophilia. However, guidelines do exist for patients with thrombophilia, and we recommend for patients with co-existing malignancies adhere to these guidelines unless specific contraindications exist or unless the risk of thromboembolism in an individual patient is deemed exceptionally high to warrant therapeutic anticoagulation.

Patients without previous thromboembolism, who have a heterozygous factor V Leiden mutation or a heterozygous prothrombin mutation G20210A are at slightly increased risk for thromboembolism as compared to those without these mutations. Therefore, these patients require routine prophylactic anticoagulation in high-risk situations, typically using prophylactic doses of LMWH. Whether DOACs are equally efficacious, has yet to be determined. However, the two studies of primary prophylactic DOAC use, AVERT, and CASSINI, enrolled over 1300 patients [17,39] and they did not exclude patients with thrombophilia. Therefore, given the prevalence of thrombophilia of at least 5% (e.g., for heterozygous factor V mutation), patients with thrombophilia were most likely included in these studies. There is no analysis of such patients in the two prophylactic studies, but a recent meta-analysis has been published on the subgroup of thrombophilia patients in the therapeutic VTE DOAC trials [114]. Breast cancer patients receiving tamoxifen treatment were found to be at increased risk for VTE if they had a heterozygous factor V Leiden mutation, and the authors recommended genetic testing for this mutation if this altered the therapeutic approach [115]. There are no general guidelines on thromboprophylaxis for these patients.

Patients with homozygous or combined (compound) factor V and/or prothrombin mutations and those with antithrombin, protein C, or protein S deficiency may require more intense VTE prophylaxis, and they often have an established regimen of prophylaxis or even therapeutic anticoagulation recommended by their local specialist. When they present with malignancy and a Khorana-Score ≥ 2 (Table 2) or other thrombogenic risk factors, a decision about VTE prophylaxis should be made (Figure 1).

Patients with antiphospholipid syndrome (APS), except for women with exclusive pregnancy-associated APS, usually require life-long therapeutic anticoagulation with vitamin K antagonists (or adjusted doses of LMWH +/- aspirin in case of necessary bridging, e.g., peri-operatively). DOACs must be avoided in patients with high-risk APS (i.e., triple-positive APS) and in those with a history of arterial thromboembolism, and it is currently also recommended to avoid them in patients with low-risk APS [116] (see current Summary of Product Characteristics for the EMA-approved DOACs).

Patients with paroxysmal nocturnal hemoglobinuria (PNH) show a high cancer incidence, and they are at very high risk for both arterial and venous thromboembolism [117]. Primary thromboprophylaxis can prevent thrombosis in these patients [118], and primary VTE prophylaxis is recommended if the PNH clone exceeds 50% of the blood cells or if patients have other risk factors (such as a co-existing malignancy) [119]. However, due to the high risk of bleeding in PNH patients and given the high efficacy of eculizumab and related drugs to decrease both the incidence of hemolysis and thrombosis, primary VTE prophylaxis in PNH patients must be balanced against the bleeding risk on an individual basis [116].

Patients with malignancies may have had previous heparin-induced thrombocytopenia (HIT) and are thus at high risk to develop VTE if subjected to heparin again. Once these patients require primary VTE prophylaxis (i.e., during immobilization or after surgery), they should receive prophylactic doses of either danaparoid (approved for HIT in EU) or fondaparinux, apixaban, or rivaroxaban (all not approved for acute HIT) [116]. All of these agents have been used in cancer patients [17,39,120], including those with HIT [121,122].

Finally, patients with Bcr-Abl negative Myeloproliferative Neoplasms (MPN), i.e., those with polycythemia vera (PV), essential thrombocythemia (ET), primary myelofibrosis (PMF), or less frequent MPN, are at increased risk for the development of venous and/or arterial thromboembolism [12,123]. Primary prophylaxis with acetylsalicylic acid (ASA) is summarized in these publications and is standard of care in all PV and intermediate- and high-risk ET patients and those with other MPN that are deemed at high risk for thromboembolic complications. While a recent analysis did not confirm the hypothesis of an elevated risk for secondary malignancies in MPN patients [124], MPN are chronic diseases, and patients may acquire a co-existing malignancy during the course of their MPN. These patients must be expected to be at excess risk for thromboembolic complications and thus require close monitoring for potential additional primary VTE prophylaxis in addition to ASA, even when they are categorized as low-risk MPN patients.

Recommendations:

- Patients with malignancies and co-existing thrombophilia require special attention and potentially alternative prophylactic anticoagulation. However, prospective data on prophylactic anticoagulation are lacking. We thus recommend adhering to guidelines for non-cancer patients with thrombophilia.
- Breast cancer patients receiving tamoxifen treatment have an increased VTE risk if they have a heterozygous factor V Leiden mutation, and we recommend genetic testing if this information is likely to change clinical management. There are no general guidelines on thromboprophylaxis in these patients.
- We recommend thromboprophylaxis in patients with cancer and homozygous or combined (compound) factor V and/or prothrombin mutations or antithrombin, protein C, or protein S deficiency if they have a Khorona-Score ≥ 2 or other thrombogenic risk factors.
- We recommend routine prophylactic anticoagulation with LMWH in high-risk situations for patients without previous thromboembolism who have a heterozygous factor V Leiden mutation or a heterozygous prothrombin mutation G20210A. Whether DOACs are equally efficacious, has yet to be determined, but they have been used in cancer patients, and the studies did not exclude patients with thrombophilia.
- Patients with thrombotic APS and cancer require long-term therapeutic anticoagulation with vitamin K antagonists or adjusted doses of LMWH +/- aspirin. DOACs must be avoided in high-risk APS (i.e., triple-positive APS) and should also be avoided in low-risk APS.
- We recommend thromboprophylaxis in patients with PNH and cancer. However, it has to be weighed against the risk of bleeding and the potential of other drugs (e.g., eculizumab) to prevent thromboembolism.
- Patients with certain Bcr-Abl negative MPN (all PV, int-/high-risk ET) should receive acetylsalicylic acid (ASA) and potentially additional primary VTE prophylaxis (individual decision for LMWH or DOAC).

### 4.7. Thromboprophylaxis in Patients with Cancer and Indwelling Catheters

Long-term central venous access devices (CVAD) such as ports are commonly used in cancer patients and have improved management of e.g., chemotherapy application, nutrition, and transfusion. On the other hand, catheter-related thrombosis (CRT) of the upper extremity is a frequent complication of such devices [125,126]. Enhanced thrombotic risk is based on several catheter-associated factors (diameter, lumen count, central vs. peripheral insertion) [127,128,129] as well as non-catheter associated factors, which are common factors in cancer patients (chemical irritation, e.g., cisplatin [130,131,132]).

The reported incidence of upper extremity thrombotic events varies from 4 to 60%. Whereas some studies showed very high rates of catheter-associated thromboembolism [133,134], other studies reported rates of about 10% or even lower [135,136,137,138,139], and differences of the incidence can be explained by e.g., heterogeneous study populations, observation times, catheter types and location, criteria of diagnosis as well as techniques to detect CRT. Whereas prophylactic anticoagulation in acutely ill hospitalized cancer patients with reduced mobility is standard of care, primary prophylaxis in the outpatient setting is still under debate (see above), even in patients with additional risk factors such as central venous catheters.

Thromboprophylaxis in patients with indwelling central venous catheters has been studied in various studies over the last decades [140,141]. Most adult studies have included patients with cancer, a cohort that is known to be at high risk of developing thromboembolic events, and drugs used for anticoagulation were VKA or LMWH [133,134,135,141,142,143]. Using warfarin in the intervention group, a benefit of thromboprophylaxis in preventing CRT was shown and confirmed in later studies using dalteparin or warfarin [133,134,143]. However, subsequent studies did not confirm these data, and early reviews, therefore, reported no benefit of prophylactic anticoagulation at all [144,145], whereas later meta-analyses of clinical trials found a slight benefit of prophylactic anticoagulation in preventing catheter-associated VTE [146,147] (Table 5).

On the other hand, the potential benefit of prophylactic anticoagulation and the risk of bleeding have to be balanced, especially in cancer patients [147]. Because of this uncertainty, most guidelines do not recommend prophylactic anticoagulation when the catheter is the sole reason for anticoagulation [155,156]. Likewise, a recent Cochrane review summarized that there is not enough evidence to recommend prophylactic anticoagulation in this setting [147]. Regarding DOACs, there are no data from randomized phase III trials regarding the effectiveness of these drugs in preventing CRT.

Recommendations:

- It is not generally recommended to administer prophylactic anticoagulation in ambulatory cancer patients solely because of implanted central venous catheters.
- The role of DOACs in this setting remains unclear.

### 4.8. Thromboprophylaxis in Patients with Multiple Myeloma

Patients with multiple myeloma (MM) or monoclonal gammopathy of undetermined significance (MGUS) have an increased risk for VTE and, to a lower degree, for arterial thromboembolic events [157,158,159,160]. The increased rate of VTE is mainly based on pathophysiologic changes by the disease and treatment-specific factors. Patients with MM have been shown to harbor higher levels of factor VIII, von Willebrand factor, and inflammatory cytokines as well as an acquired activated protein C resistance [161,162]. The rate for VTE is particularly high (up to 14–75%) for patients treated with immunomodulatory imide drugs (IMiDs), e.g., thalidomide and lenalidomide, in combination with high-dose dexamethasone (defined by at least 480 mg/month), doxorubicin, or multi-agent chemotherapy [163,164,165,166,167]. There are relatively little data regarding the VTE risk in patients treated with pomalidomide. Of note, VTE rates are higher in newly diagnosed patients (especially in the first four months) compared to patients with relapsed or refractory disease [168].

A VTE risk assessment is recommended in patients with MM, both at diagnosis and during the course of the disease. The International Myeloma Working Group (IMWG) has developed and externally validated two evidence-based VTE risk assessment models. The SAVED score can be applied in patients with MM who are on treatment with an IMiD whereas the IMPEDE VTE score is independent of IMiD therapy (Table 6) [169,170]. These scores stratify low- and high-risk patients and are incorporated in the latest NCCN guidelines (version 1.2020).

Currently, there are no randomized trials comparing different prophylaxis strategies (e.g., acetylsalicylic acid (ASA), low-molecular-weight heparin (LMWH), or warfarin) to placebo [171]. Recently performed trials in unselected myeloma patients suggest that either low-, fixed-dose warfarin (1.25 mg QD), prophylactic dose LMWH (enoxaparin 40 mg QD), or low-dose ASA (100 mg QD) lowered the incidence of VTE to less than 5% and are therefore all acceptable alternative choices for VTE prophylaxis [172,173]. Based on limited studies and the consensus opinion of experts in the field, ASA (e.g., aspirin) is recommended for patients with MM treated with IMiDs at low VTE risk, while LMWH is recommended for patients who are at high VTE risk. The VTE prophylaxis is generally administered as long as combination therapy with IMiDs is continued. LMWH may be switched to ASA after a treatment period of at least four months.

Two recently published trials investigated the prophylactic use of rivaroxaban and apixaban in cancer patients [17,39]. Patients with MM were excluded in the AVERT trial, and only 15 patients with MM were included in CASSINI [174]. In a recently published retrospective study, it was shown that apixaban in prophylactic dose (2.5 mg BID) was safe and efficacious in 56 MM patients receiving combination therapy with lenalidomide or thalidomide [175]. Currently, a number of prospective clinical trials are performed with apixaban (e.g., NCT02958969) or rivaroxaban (e.g., NCT03428373) to evaluate the safety and efficacy in the setting of primary prophylaxis in patients with MM.

Recommendations:

- VTE risk assessment is recommended in patients with MM at diagnosis and during the course of the disease.
- The SAVED score (for patients treated with IMiDs) and the IMPEDE-VTE score (independent of IMiD therapy) are recommended as validated VTE risk assessment tools and classified into low and high VTE risk. In low-risk patients, ASA or no intervention is recommended, whereas prophylaxis with LMWH is recommended in high-risk patients.
- The use of DOACs in high-risk patients is being examined in current studies.

### 4.9. Proposed Algorithm for Thromboprophylaxis in Individual Patients with Cancer

In addition to the above-mentioned clinical scenarios in which thromboprophylaxis is considered, weighing its benefits and risks, there will be numerous scenarios that cannot all be described in the present recommendations paper. Therefore, we have generated an algorithm for such situations, to provide a means for healthcare workers, including hematologists, oncologists, and internal medicine specialists but also family practitioners and nurses caring for cancer patients in their daily practice (Figure 1). This algorithm is based on the expertise of the authors and will be subject to amendments depending on the results of ongoing and future clinical trials and publications.

## 5. Conclusions

Thromboembolic events significantly contribute to morbidity and mortality in cancer patients. However, cancer patients are also at higher risk for bleeding events compared to the general population. Balancing the risk and benefit of primary pharmacological thromboprophylaxis is a daily practice for treating physicians, especially in patients during oncologic treatment. Defining the optimal approach is often difficult, with the number of anti-cancer drugs with elevated bleeding or VTE risks increasing, as well as emerging orally applied drugs for prophylactic anticoagulation in addition to parenterally used heparins. Therefore, we aimed to analyze current treatment options with regard to individual VTE-risk by reviewing the literature and discuss controversial topics among an expert panel of hematologists, oncologists, and hemostasis specialists to provide assistance in the decision-making process whether to initiate primary pharmacological prophylaxis in cancer patients or not. We have designed an algorithm to help with such decisions in light of the current absence of data from randomized trials in many clinical scenarios. This algorithm will be open for adjustments in the future when such evidence becomes available.

## Figures and Tables

**Table 1:** Selection of randomized clinical trials investigating the role of pharmacological thromboprophylaxis in cancer patients undergoing anti-cancer therapy.

Name of the Study and Reference	*n*	Patient Cohorts (Cancer Type or Khorana Risk)	1°EP	1°EP Control	1°EP Experimental	RRR	ARR
PROTECHT [37]	1150	Mixed	sVTE, sArtE.	Placebo 3.9%	Nadroparin (3800 U QD) 2.0%	49%	1.9%
SAVE-ONCO [23]	3212	Mixed	sDVT, PE, VTE-rD	Placebo 3.4%	Semuloparin (20 mg QD) 1.2%	65%	2.2%
PRH-HCTU-FRAGEM [26]	123	Pancreatic cancer	s/i VTE, s/i ArtE	Observation 23%	Dalteparin (200 IU ≥ 150/kg/d) 3.4%	85%	19.6%
CONKO-004 [22]	312	Pancreatic cancer	sVTE	Observation 10.2%	Enoxaparin (1 mg/kg/d) 1.3%	87%	8.9%
RASTEN [38]	377	Small cell lung cancer	sVTE	Observation 8.4%	Enoxaparin (1 mg/kg/d) 2.7%	71%	5.7%
PHACS [18]	117	Khorana Score ≥3	all VTE	Observation 21%	Dalteparin (5000 U/d) 12%	43%	9.0%
CASSINI [39]	841	Khorana Score ≥2	s/i VTE, VTE-rD	Placebo 8.8%	Rivaroxaban (10 mg QD) 6.0%	32%	2.8%
AVERT [17]	563	Khorana Score ≥2	s/i VTE, VTE-rD	Placebo 10.2%	Apixaban 2.5 mg BID 4.2%	59%	6.0%

Abbreviations: ARR = absolute risk reduction, BID = twice daily, 1°EP = primary endpoint of the studies, QD = once daily, RRR = relative risk reduction, s/i ArtE = symptomatic/incidental arterial event, sDVT = symptomatic deep vein thrombosis, s/i VTE = symptomatic/incidental venous thromboembolism, VTE-rD = VTE-related death.

**Table 3:** Anticoagulant indications, doses, and dose adjustments for kidney dysfunction *.

Anticoagulant	Relevant Indications	Dose	Dose Adjustment for Kidney Dysfunction (See Label for More Information)
Unfractionated heparin (UFH)	Prophylaxis of venous or arterial thromboembolism	Prophylaxis: 5000 IE s.c. q8–12 h or 7500 IE s.c. q12 h	Exert use possible in renal insufficiency, but requires close monitoring
Enoxaparin	Prophylaxis of VTE in surgical pts with moderate and high VTE risk Prophylaxis of VTE in pts with reduced mobility	Prophylaxis mod. risk: 20 mg s.c. QD Prophylaxis high risk: 40 mg s.c. QD	Mild to moderate kidney dysfunction (creatinine clearance >/= 30 mL/min): No dose adjustment, but careful clinical observation Severe kidney dysfunction (creatinine clearance 15–30 mL/min): Prophylaxis 20 mg s.c. QD Kidney failure (creatinine clearance < 15 mL/min): Use not recommended
Dalteparin	Peri- and postoperative primary prophylaxis of DVT in pts with low/mod. VTE risk Peri- and postoperative primary prophylaxis of DVT in pts with high VTE risk Primary prophylaxis of DVT in pts with medical conditions (internal medicine) in pts with mod./high VTE risk and transient immobility	Prophylaxis low/mod. risk: 2500 IE s.c. QD Prophylaxis high risk: 2500–5000 IE s.c. QD (see label) Prophylaxis medical: 5000 IE s.c. QD	Kidney failure (creatinine clearance < 15 mL/min): Use only with close observation
Certoparin	Prophylaxis of VTE	3000 IE s.c. QD	Severe kidney dysfunction (creatinine clearance </= 30 mL/min): Great care is necessary
Nadroparin	Prophylaxis of VTE	Prophylaxis: 2850–5700 IE s.c. QD	Mild to moderate kidney dysfunction (creatinine clearance 30–80 mL/min): Use with great care, monitor anti-Xa levels Severe kidney dysfunction (creatinine clearance </= 30 mL/min): Use is contraindicated
Reviparin	Prophylaxis of VTE	Prophylaxis: 1750 IE s.c. QD	Mild to moderate kidney dysfunction (creatinine clearance 30–80 mL/min): Use with great care, monitor anti-Xa levels Severe kidney dysfunction (creatinine clearance </= 30 mL/min): Use is contraindicated
Fondaparinux	Prophylaxis of VTE	Prophylaxis: 2.5 mg s.c. QD	Moderate kidney dysfunction (creatinine clearance 30–50 mL/min): Use with great care Severe kidney dysfunction (creatinine clearance </= 30 mL/min): Use is contraindicated
Apixaban	Prophylaxis of VTE (off-label) *	Prophylaxis: 2.5 mg BID	Moderate kidney dysfunction (creatinine clearance 30–60 mL/min): no dose reduction Severe kidney dysfunction (creatinine clearance </= 30 mL/min): Use with great care Kidney failure (creatinine clearance < 15 mL/min): Use not recommended
Rivaroxaban	Prophylaxis of VTE (off-label) *	Prophylaxis: 10 mg QD	Moderate kidney dysfunction (creatinine clearance 30–60 mL/min): no dose reduction Severe kidney dysfunction (creatinine clearance </= 30 mL/min): Use with great care Kidney failure (creatinine clearance < 15 mL/min): Use not recommended

* DOACs are not approved for thromboprophylaxis besides post-surgery in Europe, and, therefore, dosing in general and dose adjustments for kidney dysfunction are based on recommendations in post-surgery patients.

**Table 4:** Types of thrombophilia [113].

Hereditary	Acquired
Factor V Leiden mutation • heterozygous • homozygous	Antiphospholipid syndrome
Prothrombin mutation • heterozygous • homozygous	Medication-induced thrombophilia (see also sections below) • Lenalidomide, thalidomide, pomalidomide(?), all particularly in combination with corticosteroids) (see the section on multiple myeloma below) • Estrogens • Heparin-induced thrombocytopenia (HIT)
Antithrombin deficiency	Paroxysmal nocturnal hemoglobinuria (PNH)
Protein C deficiency	Myeloproliferative neoplasms (e.g., PV, ET)
Protein S deficiency	
Combined thrombophilias (e.g., factor V and prothrombin mutations)	

Abbreviations: PV = Polycythemia vera; ET = Essential thrombocythemia.

**Table 5:** Studies for prophylaxis of central venous device-related thrombosis (CRT).

Author	Year	*n*	Intervention	PE	PE Control	PE Experimental	*p*-Value
Bern et al. [133]	1990	121	VKA vs. placebo	DVT (VG)	37.5%	9.5%	<0.001
Monreal et al. [134]	1996	29	LMWH vs. placebo	DVT (VG)	62%	6%	0.002
Boraks et al. [143]	1998	223	VKA vs. placebo	DVT (US, VG)	13%	5%	0.03
Heaton et al. [142]	2002	88	VKA vs. placebo	DVT (VG)	11.6%	17.8%	0.42
Mismetti et al. [148]	2003	59	VKA (v), LMWH (h)	DVT (VG)	16.7% (v)	28.6% (h)	0.48
Couban et al. [149]	2005	255	VKA vs. placebo	DVT (symptomatic)	4.6%	4.0%	ns
Ruud et al. * [150]	2006	73	VKA vs. placebo	DVT	36%	48%	0.44
Karthaus et al. [138]	2006	439	LMWH vs. placebo	DVT (VG or US)	3.4%	3.7%	0.83
Verso et al. [151]	2008	385	LMWH vs. placebo	DVT (VG)	18%	14.1%	0.35
De Cicco et al. [152]	2009	450	VKA (v), LWMH (h), placebo	DVT (VG)	52.6%	21.5%(v)/40% (h)	<0.01 (w); 0.05 (d)
Young et al. [153]	2009	812	VKAvs placebo	DVT (symptomatic)	6%	6%	0.98
Lavau-Denes et al. [137]	2013	420	VKA or LMWH vs. placebo	DVT	14.8%	8%	0.0357
Niers et al. [154]	2013	113	VKA vs. placebo	DVT (VG)	9%	17%	0.49

VKA = Vitamin K Antagonists, LMWH = low molecular weight heparin, PE = primary endpoint, US = ultrasound, VG = venography, DVT = deep vein thrombosis. * study in pediatric patients.

**Table 6:** IMPEDE-VTE Score, adapted from [170].

Variable	Point Score
IMiD therapy	+4
BMI ≥25 kg/m^2^	+1
Pelvic, hip or femur fracture	+4
Erythropoiesis-stimulating agent	+1
Dexamethason (regimen dose)	
– Standard dose (≤160 mg/month) – High dose (>160 mg/month)	+2 +4
Doxorubicin	+3
Ethnicity/Race = Asian/Pacific Islander	-3
History of VTE before multiple myeloma diagnosis	+5
Tunneled line or central venous catheter	+2
Existing thromboprophylaxis: therapeutic LMWH or Warfarin	−4
Existing thromboprophylaxis: prophylactic LMWH or aspirin	−3

Calculate risk: Recommendation for high risk (>3 points): LMWH (dose equivalent to enoxaparin 40 mg once daily); Recommendation for low risk (≤3 points): no intervention or Aspirin 100 mg once daily.

## Data Availability

Since this is a review article, please refer to the extensive reference list for all available data regarding this article.

## Supplementary Materials

The following are available online at https://www.mdpi.com/article/10.3390/cancers13122905/s1, Table S1: Combined presentation of all recommendations sorted by chapter.

## References

[1] Khorana A.A., Francis C.W., Culakova E., Kuderer N.M., Lyman G.H. (2007). Thromboembolism is a leading cause of death in cancer patients receiving outpatient chemotherapy. J. Thromb. Haemost..

[2] Sørensen H.T., Mellemkjær L., Olsen J.H., Baron J.A. (2000). Prognosis of Cancers Associated with Venous Thromboembolism. N. Engl. J. Med..

[3] Fuentes H.E., Tafur A.J., Caprini J.A., Alatri A., Trujillo-Santos J., Farge-Bancel D., Rosa V., Font C., Vilaseca A., Monreal M. (2019). Prediction of early mortality in patients with cancer-associated thrombosis in the RIETE Database. Int. Angiol..

[4] Fuentes H.E., Oramas D.M., Paz L.H., Wang Y., Andrade X.A., Tafur A.J. (2018). Venous Thromboembolism Is an Independent Predictor of Mortality Among Patients with Gastric Cancer. J. Gastrointest. Cancer.

[5] de Meis E., Pinheiro V.R., Zamboni M.M., Guedes M.T., Castilho I.A., Martinez M.M., Leda M.S., Silveira N.P., Rumjanek V.M., Levy R.A. (2009). Clotting, immune system, and venous thrombosis in lung adenocarcinoma patients: A prospective study. Cancer Investig..

[6] Fuentes H., Oramas D., Paz L., Casanegra A., Mansfield A., Tafur A. (2017). Meta-analysis on anticoagulation and prevention of thrombosis and mortality among patients with lung cancer. Thromb. Res..

[7] Chew H.K., Wun T., Harvey D.J., Zhou H., White R.H. (2007). Incidence of Venous Thromboembolism and the Impact on Survival in Breast Cancer Patients. J. Clin. Oncol..

[8] Chaturvedi S., Sidana S., Elson P., Khorana A.A., McCrae K.R. (2014). Symptomatic and Incidental Venous Thromboembolic Disease Are Both Associated with Mortality in Patients with Prostate Cancer. PLoS ONE.

[9] Mahajan A., Wun T., Chew H., White R.H. (2014). Lymphoma and venous thromboembolism: Influence on mortality. Thromb. Res..

[10] Kuderer N.M., Ortel T.L., Francis C.W. (2009). Impact of Venous Thromboembolism and Anticoagulation on Cancer and Cancer Survival. J. Clin. Oncol..

[11] Heit J.A., Silverstein M.D., Mohr D.N., Petterson T.M., O'Fallon W.M., Melton L.J. (2000). Risk factors for deep vein thrombosis and pulmonary embolism: A population-based case-control study. Arch. Intern. Med..

[12] Barbui T., Finazzi G., Falanga A. (2013). Myeloproliferative neoplasms and thrombosis. Blood.

[13] Ahlbrecht J., Dickmann B., Ay C., Dunkler D., Thaler J., Schmidinger M., Quehenberger P., Haitel A., Zielinski C., Pabinger I. (2012). Tumor Grade Is Associated With Venous Thromboembolism in Patients With Cancer: Results From the Vienna Cancer and Thrombosis Study. J. Clin. Oncol..

[14] Khorana A.A., Dalal M., Lin J., Connolly G.C. (2012). Incidence and predictors of venous thromboembolism (VTE) among ambulatory high-risk cancer patients undergoing chemotherapy in the United States. Cancer.

[15] Zhou H., Romano P.S., White R.H. (2003). Incidence of symptomatic venous thromboembolism after different elective or urgent surgical procedures. Thromb. Haemost..

[16] Carrier M., Khorana A.A., Moretto P., Le Gal G., Karp R., Zwicker J.I. (2014). Lack of Evidence to Support Thromboprophylaxis in Hospitalized Medical Patients with Cancer. Am. J. Med..

[17] Carrier M., Abou-Nassar K., Mallick R., Tagalakis V., Shivakumar S., Schattner A., Kuruvilla P., Hill D., Spadafora S., Marquis K. (2019). Apixaban to Prevent Venous Thromboembolism in Patients with Cancer. N. Engl. J. Med..

[18] Khorana A.A., Francis C.W., Kuderer N.M., Carrier M., Ortel T.L., Wun T., Rubens D., Hobbs S., Iyer R., Peterson D. (2017). Dalteparin thromboprophylaxis in cancer patients at high risk for venous thromboembolism: A randomized trial. Thromb. Res..

[19] Schünemann H.J., Ventresca M., Crowther M., Briel M., Zhou Q., Garcia D., Lyman G., Noble S., Macbeth F., Griffiths G. (2016). Use of heparins in patients with cancer: Individual participant data meta-analysis of randomised trials study protocol. BMJ Open.

[20] Schünemann H.J., Ventresca M., Crowther M., Briel M., Zhou Q., Noble S., Macbeth F., Griffiths G., Garcia D., Lyman G.H. (2020). Evaluating prophylactic heparin in ambulatory patients with solid tumours: A systematic review and individual participant data meta-analysis. Lancet Haematol..

[21] Xin Z., Liu F., Du Y., Mao F., Wang X., Xu P., Li Z., Qian J., Yao J. (2020). Primary prophylaxis for venous thromboembolism in ambulatory cancer patients: A systematic review and network meta-analysis. Ann. Palliat. Med..

[22] Pelzer U., Opitz B., Deutschinoff G., Stauch M., Reitzig P.C., Hahnfeld S., Müller L., Grunewald M., Stieler J.M., Sinn M. (2015). Efficacy of Prophylactic Low–Molecular Weight Heparin for Ambulatory Patients With Advanced Pancreatic Cancer: Outcomes From the CONKO-004 Trial. J. Clin. Oncol..

[23] Agnelli G., George D.J., Kakkar A.K., Fisher W., Lassen M.R., Mismetti P., Mouret P., Chaudhari U., Lawson F., Turpie A.G. (2012). Semuloparin for Thromboprophylaxis in Patients Receiving Chemotherapy for Cancer. N. Engl. J. Med..

[24] Kakkar V.V., Balibrea J.L., Martínez-González J., Prandoni P. (2010). Extended prophylaxis with bemiparin for the prevention of venous thromboembolism after abdominal or pelvic surgery for cancer: The CANBESURE randomized study. J. Thromb. Haemost..

[25] Agnelli G., Verso M. (2010). Thromboprophylaxis during chemotherapy in patients with advanced cancer. Thromb. Res..

[26] Maraveyas A., Waters J., Roy R., Fyfe D., Propper D., Lofts F., Sgouros J., Gardiner E., Wedgwood K., Ettelaie C. (2012). Gemcitabine versus gemcitabine plus dalteparin thromboprophylaxis in pancreatic cancer. Eur. J. Cancer.

[27] Samama M.M., Cohen A.T., Darmon J.-Y., Desjardins L., Eldor A., Janbon C., Leizorovicz A., Nguyen H., Olsson C.-G., Turpie A.G. (1999). A Comparison of Enoxaparin with Placebo for the Prevention of Venous Thromboembolism in Acutely Ill Medical Patients. N. Engl. J. Med..

[28] Leizorovicz A., Cohen A.T., Turpie A.G., Olsson C.-G., Vaitkus P.T., Goldhaber S.Z. (2004). Randomized, Placebo-Controlled Trial of Dalteparin for the Prevention of Venous Thromboembolism in Acutely Ill Medical Patients. Circ..

[29] Cohen A.T., Davidson B.L., Gallus A.S., Lassen M.R., Prins M.H., Tomkowski W., Turpie A.G.G., Egberts J.F.M., Lensing A.W.A. (2006). Efficacy and safety of fondaparinux for the prevention of venous thromboembolism in older acute medical patients: Randomised placebo controlled trial. BMJ.

[30] Riess H., Haas S., Tebbe U., Gerlach H.-E., Abletshauser C., Sieder C., Rossol S., Pfeiffer B., Schellong S.M. (2010). A randomized, double-blind study of certoparin vs. unfractionated heparin to prevent venous thromboembolic events in acutely ill, non-surgical patients: CERTIFY Study. J. Thromb. Haemost..

[31] Haas S., Schellong S.M., Tebbe U., Gerlach H.-E., Bauersachs R., Melzer N., Abletshauser C., Sieder C., Bramlage P., Riess H. (2011). Heparin based prophylaxis to prevent venous thromboembolic events and death in patients with cancer—A subgroup analysis of CERTIFY. BMC Cancer.

[32] Lyman G.H., Bohlke K., Khorana A.A., Kuderer N.M., Lee A.Y., Arcelus J.I., Balaban E.P., Clarke J.M., Flowers C.R., Francis C.W. (2015). Venous Thromboembolism Prophylaxis and Treatment in Patients With Cancer: American Society of Clinical Oncology Clinical Practice Guideline Update 2014. J. Clin. Oncol..

[33] Mandalà M., Labianca R. (2010). Venous thromboembolism (VTE) in cancer patients. ESMO Clinical Recommendations for prevention and management. Thromb. Res..

[34] S3-Leitlinie Prophylaxe der Venösen Thromboembolie (VTE). https://www.awmf.org/uploads/tx_szleitlinien/003-001l_S3_VTE-Prophylaxe_2015-12.pdf.

[35] Riess H., Pabinger-Fasching I., Alt-Epping B., Demarmels Biasiutti F., Langer F., Wörmann B. Venöse Thrombembolien (VTE) bei Tumorpatienten. https://www.onkopedia.com/de/onkopedia/guidelines/venoese-thrombembolien-vte-bei-tumorpatienten/@@guideline/html/index.html.

[36] Patell R., Rybicki L., McCrae K.R., Khorana A.A. (2017). Predicting risk of venous thromboembolism in hospitalized cancer patients: Utility of a risk assessment tool. Am. J. Hematol..

[37] Agnelli G., Gussoni G., Bianchini C., Verso M., Mandalà M., Cavanna L., Barni S., Labianca R., Buzzi F., Scambia G. (2009). Nadroparin for the prevention of thromboembolic events in ambulatory patients with metastatic or locally advanced solid cancer receiving chemotherapy: A randomised, placebo-controlled, double-blind study. Lancet Oncol..

[38] Ek L., Gezelius E., Bergman B., Bendahl P., Anderson H., Sundberg J., Wallberg M., Falkmer U., Verma S., Belting M. (2018). Randomized phase III trial of low-molecular-weight heparin enoxaparin in addition to standard treatment in small-cell lung cancer: The RASTEN trial. Ann. Oncol..

[39] Khorana A.A., Soff G.A., Kakkar A.K., Vadhan-Raj S., Riess H., Wun T., Streiff M.B., Garcia D.A., Liebman H.A., Belani C.P. (2019). Rivaroxaban for Thromboprophylaxis in High-Risk Ambulatory Patients with Cancer. N. Engl. J. Med..

[40] Cohen A.T., Katholing A., Rietbrock S., Bamber L., Martinez C. (2017). Epidemiology of first and recurrent venous thromboembolism in patients with active cancer. A population-based cohort study. Thromb. Haemost..

[41] Di Nisio M., Porreca E., Candeloro M., De Tursi M., Russi I., Rutjes A.W. (2016). Primary prophylaxis for venous thromboembolism in ambulatory cancer patients receiving chemotherapy. Cochrane Database Syst. Rev..

[42] Akl E.A., Kahale L.A., Ballout R.A., Barba M., Yosuico V.E.D., Van Doormaal F.F., Middeldorp S., Bryant A., Schünemann H. (2014). Parenteral anticoagulation in ambulatory patients with cancer. Cochrane Database Syst. Rev..

[43] Meyer G., Besse B., Doubre H., Charles-Nelson A., Aquilanti S., Izadifar A., Azarian R., Monnet I., Lamour C., Descourt R. (2018). Anti-tumour effect of low molecular weight heparin in localised lung cancer: A phase III clinical trial. Eur. Respir. J..

[44] Khorana A.A., Kuderer N.M., Culakova E., Lyman G.H., Francis C.W. (2008). Development and validation of a predictive model for chemotherapy-associated thrombosis. Blood.

[45] Ay C., Dunkler D., Marosi C., Chiriac A.-L., Vormittag R., Simanek R., Quehenberger P., Zielinski C., Pabinger I. (2010). Prediction of venous thromboembolism in cancer patients. Blood.

[46] Carrier M., Le Gal G., Wells P.S. (2019). Preventing Venous Thromboembolism in Patients with Cancer. Reply. N. Engl. J. Med..

[47] Kumar V., Shaw J.R., Key N.S., Ilich A., Mallick R., Wells P.S., Carrier M. (2020). D-Dimer Enhances Risk-Targeted Thromboprophylaxis in Ambulatory Patients with Cancer. Oncologist.

[48] Pabinger I., van Es N., Heinze G., Posch F., Riedl J., Reitter E.-M., Di Nisio M., Cesarman-Maus G., Kraaijpoel N., Zielinski C.C. (2018). A clinical prediction model for cancer-associated venous thromboembolism: A development and validation study in two independent prospective cohorts. Lancet Haematol..

[49] Schulman S., Kearon C., The Subcommittee on Control of Anticoagulation of the Scientific and Standardization Committee Of the International Society on Thrombosis and Haemostasis (2005). Definition of major bleeding in clinical investigations of antihemostatic medicinal products in non-surgical patients. J. Thromb. Haemost..

[50] Mansfield A.S., Tafur A.J., Wang C.E., Kourelis T.V., Wysokinska E.M., Yang P. (2016). Predictors of active cancer thromboembolic outcomes: Validation of the Khorana score among patients with lung cancer. J. Thromb. Haemost..

[51] Preissner S., Kroll K., Dunkel M., Senger C., Goldsobel G., Kuzman D., Guenther S., Winnenburg R., Schroeder M., Preissner R. (2009). SuperCYP: A comprehensive database on Cytochrome P450 enzymes including a tool for analysis of CYP-drug interactions. Nucleic Acids Res..

[52] Kim S.-A., Yhim H.-Y., Bang S.-M. (2019). Current Management of Cancer-associated Venous Thromboembolism: Focus on Direct Oral Anticoagulants. J. Korean Med. Sci..

[53] Vazquez S.R. (2018). Drug-drug interactions in an era of multiple anticoagulants: A focus on clinically relevant drug interactions. Blood.

[54] Hakeam H.A., Al-Sanea N. (2017). Effect of major gastrointestinal tract surgery on the absorption and efficacy of direct acting oral anticoagulants (DOACs). J. Thromb. Thrombolysis.

[55] Song Y., Wang X., Perlstein I., Wang J., Badawy S., Frost C., LaCreta F. (2015). Relative Bioavailability of Apixaban Solution or Crushed Tablet Formulations Administered by Mouth or Nasogastric Tube in Healthy Subjects. Clin. Ther..

[56] Khorana A.A., Francis C.W., Culakova E., Kuderer N.M., Lyman G.H. (2007). Frequency, risk factors, and trends for venous thromboembolism among hospitalized cancer patients. Cancer.

[57] Cronin M., Dengler N., Krauss E.S., Segal A., Wei N., Daly M., Mota F., Caprini J.A. (2019). Completion of the Updated Caprini Risk Assessment Model (2013 Version). Clin. Appl. Thromb..

[58] Clagett G., Reisch J. (1989). Prevention of Venous Thromboembolism in General, Surgical Patients: Results of a Meta-Analysis. J. Urol..

[59] Akl E.A., Labedi N., Terrenato I., Barba M., Sperati F., Sempos E.V., Muti P., Cook D., Schünemann H. (2011). Low molecular weight heparin versus unfractionated heparin for perioperative thromboprophylaxis in patients with cancer. Cochrane Database Syst. Rev..

[60] Wang T.-F., Li A., Garcia D. (2018). Managing thrombosis in cancer patients. Res. Pr. Thromb. Haemost..

[61] Bergqvist D., Agnelli G., Cohen A.T., Eldor A., Nilsson P.E., Le Moigne-Amrani A., Dietrich-Neto F. (2002). Duration of Prophylaxis against Venous Thromboembolism with Enoxaparin after Surgery for Cancer. N. Engl. J. Med..

[62] Rasmussen M.S., Jorgensen L.N., Wille-Jørgensen P. (2009). Prolonged thromboprophylaxis with Low Molecular Weight heparin for abdominal or pelvic surgery. Cochrane Database Syst. Rev..

[63] Felder S., Rasmussen M.S., King R., Sklow B., Kwaan M., Madoff R., Jensen C. (2019). Prolonged thromboprophylaxis with low molecular weight heparin for abdominal or pelvic surgery. Cochrane Database Syst. Rev..

[64] Akl E.A., Terrenato I., Barba M., Sperati F., Muti P., Schünemann H.J. (2008). Extended perioperative thromboprophylaxis in patients with cancer. A systematic review. Thromb. Haemost..

[65] Bottaro F.J., Elizondo M.C., Doti C., Bruetman J.E., Perez Moreno P.D., Bullorsky E.O., Ceresetto J.M. (2008). Efficacy of extended thrombo-prophylaxis in major abdominal surgery: What does the evidence show? A meta-analysis. Thromb. Haemost..

[66] Rasmussen M.S., Jorgensen L.N., Wille-Jørgensen P., Nielsen J.D., Horn A., Mohn A.C., Sømod L., Olsen B. (2006). Prolonged prophylaxis with dalteparin to prevent late thromboembolic complications in patients undergoing major abdominal surgery: A multicenter randomized open-label study. J. Throm. Haemost..

[67] Guo Q., Huang B., Zhao J., Ma Y., Yuan D., Yang Y., Du X. (2017). Perioperative Pharmacological Thromboprophylaxis in Patients With Cancer: A Systematic Review and Meta-analysis. Ann. Surg..

[68] Fagarasanu A., Alotaibi G.S., Hrimiuc R., Lee A.Y.Y., Wu C. (2016). Role of Extended Thromboprophylaxis After Abdominal and Pelvic Surgery in Cancer Patients: A Systematic Review and Meta-Analysis. Ann. Surg. Oncol..

[69] Guyatt G.H., Akl E.A., Crowther M., Gutterman D.D., Schuünemann H.J. (2012). Executive summary: Antithrombotic Therapy and Prevention of Thrombosis, 9th ed: American College of Chest Physicians Evidence-Based Clinical Practice Guidelines. Chest.

[70] Khorana A.A., Carrier M., Garcia D.A., Lee A.Y.Y. (2016). Guidance for the prevention and treatment of cancer-associated venous thromboembolism. J. Thromb. Thrombolysis.

[71] Kitchens C.S., Alving B.M., Kessler C.M. (2007). Consultative Hemostasis and Thrombosis.

[72] Schwahn-Schreiber C., Breu F.X., Rabe E., Buschmann I., Döller W., Lulay G.R., Miller A., Valesky E., Reich-Schupke S. (2018). S1 guideline on intermittent pneumatic compression (IPC). Der Hautarzt Zeitschrift Dermatologie Venerologie Verwandte Gebiete.

[73] Launay-Vacher V., Oudard S., Janus N., Gligorov J., Pourrat X., Rixe O., Morere J.F., Beuzeboc P., Deray G. (2007). Prevalence of Renal Insufficiency in cancer patients and implications for anticancer drug management: The renal insufficiency and anticancer medications (IRMA) study. Cancer.

[74] Mahmoodi B.K., Gansevoort R.T., Naess I.A., Lutsey P.L., Braekkan S.K., Veeger N.J., Brodin E.E., Meijer K., Sang Y., Matsushita K. (2012). Association of mild to moderate chronic kidney disease with venous thromboembolism: Pooled analysis of five prospective general population cohorts. Circulation.

[75] Nishimoto Y., Yamashita Y., Kim K., Morimoto T., Saga S., Amano H., Takase T., Hiramori S., Oi M., Akao M. (2020). Risk Factors for Major Bleeding During Anticoagulation Therapy in Cancer-Associated Venous Thromboembolism—From the COMMAND VTE Registry. Circ. J..

[76] Pai M., Adhikari N.K.J., Ostermann M., Heels-Ansdell D., Douketis J.D., Skrobik Y., Qushmaq I., Meade M., Guyatt G., Geerts W. (2018). Low-molecular-weight heparin venous thromboprophylaxis in critically ill patients with renal dysfunction: A subgroup analysis of the PROTECT trial. PLoS ONE.

[77] Chan K.E., Thadhani R.I., Maddux F.W. (2013). No difference in bleeding risk between subcutaneous enoxaparin and heparin for thromboprophylaxis in end-stage renal disease. Kidney Int..

[78] Hughes S., Szeki I., Nash M.J., Thachil J. (2014). Anticoagulation in chronic kidney disease patients--the practical aspects. Clin. Kidney J..

[79] Pimpin L., Cortez-Pinto H., Negro F., Corbould E., Lazarus J., Webber L., Sheron N. (2018). Burden of liver disease in Europe: Epidemiology and analysis of risk factors to identify prevention policies. J. Hepatol..

[80] Bunchorntavakul C., Reddy K.R. (2017). Drug Hepatotoxicity: Newer Agents. Clin. Liver Dis..

[81] Khoury T., Ayman A.R., Cohen J., Daher S., Shmuel C., Mizrahi M. (2016). The Complex Role of Anticoagulation in Cirrhosis: An Updated Review of Where We Are and Where We Are Going. Digestion.

[82] Tripodi A., Mannucci P.M. (2011). The Coagulopathy of Chronic Liver Disease. N. Engl. J. Med..

[83] Ogren M., Bergqvist D., Bjorck M., Acosta S., Eriksson H., Sternby N.H. (2006). Portal vein thrombosis: Prevalence, patient characteristics and lifetime risk: A population study based on 23,796 consecutive autopsies. World J. Gastroenterol..

[84] Rodriguez-Castro K.I., Simioni P., Burra P., Senzolo M. (2012). Anticoagulation for the treatment of thrombotic complications in patients with cirrhosis. Liver Int..

[85] Villa E., Cammà C., Marietta M., Luongo M., Critelli R., Colopi S., Tata C., Zecchini R., Gitto S., Petta S. (2012). Enoxaparin Prevents Portal Vein Thrombosis and Liver Decompensation in Patients With Advanced Cirrhosis. Gastroenterology.

[86] Steffel J., Verhamme P., Potpara T.S., Albaladejo P., Antz M., Desteghe L., Haeusler K.G., Oldgren J., Reinecke H., Roldan-Schilling V. (2018). The 2018 European Heart Rhythm Association Practical Guide on the use of non-vitamin K antagonist oral anticoagulants in patients with atrial fibrillation. Eur. Heart J..

[87] Liakoni E., Bravo A.E.R., Terracciano L., Heim M., Krähenbühl S. (2014). Symptomatic Hepatocellular Liver Injury With Hyperbilirubinemia in Two Patients Treated With Rivaroxaban. JAMA Intern. Med..

[88] Anastasia E.J., Rosenstein R.S., Bergsman J.A., Parra D. (2015). Use of apixaban after development of suspected rivaroxaban-induced hepatic steatosis; a case report. Blood Coagul. Fibrinolysis.

[89] Rossel A., Robert-Ebadi H., Combescure C., Grosgurin O., Stirnemann J., Addeo A., Garin N., Agoritsas T., Reny J.-L., Marti C. (2019). Anticoagulant therapy for acute venous thrombo-embolism in cancer patients: A systematic review and network meta-analysis. PLoS ONE.

[90] Key N.S., Khorana A.A., Kuderer N.M., Bohlke K., Lee A.Y., Arcelus J.I., Wong S.L., Balaban E.P., Flowers C.R., Francis C.W. (2020). Venous Thromboembolism Prophylaxis and Treatment in Patients With Cancer: ASCO Clinical Practice Guideline Update. J. Clin. Oncol..

[91] Wang Y., Lv H., Li D., Chen C., Gu G., Sun Y., Yang X., Liu Y., Fang F., Liu J. (2019). Efficacy and Safety of Direct Oral Anticoagulants for Secondary Prevention of Cancer-Associated Thrombosis: A Systematic Review and Meta-Analysis of Randomized Controlled Trials and Prospective Cohort Studies. Front. Pharmacol..

[92] Frere C., Benzidia I., Marjanovic Z., Farge D. (2019). Recent Advances in the Management of Cancer-Associated Thrombosis: New Hopes but New Challenges. Cancers.

[93] Schlichtig K., Dürr P., Dörje F., Fromm M.F. (2019). New Oral Anti-Cancer Drugs and Medication Safety. Dtsch. Aerzteblatt Online.

[94] Brandes A., Scelzi E., Salmistraro G., Ermani M., Carollo C., Berti F., Zampieri P., Baiocchi C., Fiorentino M. (1997). Incidence and risk of thromboembolism during treatment of high-grade gliomas: A prospective study. Eur. J. Cancer.

[95] Yust-Katz S., Mandel J.J., Wu J., Yuan Y., Webre C., Pawar T.A., Lhadha H.S., Gilbert M.R., Armstrong T.S. (2015). Venous thromboembolism (VTE) and glioblastoma. J. Neuro-Oncol..

[96] Dickinson L.D., Miller L.D., Patel C.P., Gupta S.K. (1998). Enoxaparin Increases the Incidence of Postoperative Intracranial Hemorrhage when Initiated Preoperatively for Deep Venous Thrombosis Prophylaxis in Patients with Brain Tumors. Neurosurgery.

[97] Perry J.R., Julian J.A., Laperriere N.J., Geerts W., Agnelli G., Rogers L.R., Malkin M.G., Sawaya R., Baker R., Falanga A. (2010). PRODIGE: A randomized placebo-controlled trial of dalteparin low-molecular-weight heparin thromboprophylaxis in patients with newly diagnosed malignant glioma. J. Thromb. Haemost..

[98] Taillibert S., Taillandier L., Le Rhun E. (2015). Venous thrombosis in patients with high-grade glioma. Curr. Opin. Oncol..

[99] Ay C., Kamphuisen P., Agnelli G. (2017). Antithrombotic therapy for prophylaxis and treatment of venous thromboembolism in patients with cancer: Review of the literature on current practice and emerging options. ESMO Open.

[100] Samuelson Bannow B.T., Lee A., Khorana A.A., Zwicker J.I., Noble S., Ay C., Carrier M. (2018). Management of cancer-associated thrombosis in patients with thrombocytopenia: Guidance from the SSC of the ISTH. J. Thromb. Haemost..

[101] Samuelson Bannow B.T., Walter R.B., Gernsheimer T.B., Garcia D.A. (2017). Patients treated for acute VTE during periods of treatment-related thrombocytopenia have high rates of recurrent thrombosis and transfusion-related adverse outcomes. J. Thromb. Thrombolysis.

[102] Cortelezzi A., Moia M., Falanga A., Pogliani E.M., Agnelli G., Bonizzoni E., Gussoni G., Barbui T., Mannucci P.M. (2005). Incidence of thrombotic complications in patients with haematological malignancies with central venous catheters: A prospective multicentre study. Br. J. Haematol..

[103] Easaw J.C., Shea-Budgell M.A., Wu C.M., Czaykowski P.M., Kassis J., Kuehl B., Lim H.J., MacNeil M., Martinusen D., McFarlane P.A. (2015). Canadian consensus recommendations on the management of venous thromboembolism in patients with cancer. Part 1: Prophylaxis. Curr. Oncol..

[104] Herishanu Y., Misgav M., Kirgner I., Ben-Tal O., Eldor A., Naparstek E. (2004). Enoxaparin can be used safely in patients with severe thrombocytopenia due to intensive chemotherapy regimens. Leuk. Lymphoma.

[105] Chalayer E., Cavalieri D., Martignoles J.A., Genthon A., Tavernier E., Tardy B. (2017). Antithrombotic therapy and platelet transfusions in hematologic malignancy patients presenting chemotherapy-induced thrombocytopenia: A French survey. Transfusion.

[106] Pabinger-Fasching I., Alt-Epping B., Demarmels Biasiutti F., Langer F., Riess H., Wörrmann B. Thrombembolien (VTE) bei Tumorpatienten. https://www.onkopedia.com/de/onkopedia/archive/guidelines/venoese-thrombembolien-vte-bei-tumorpatienten/version-09042019T082542/@@guideline/html/index.html.

[107] Watson H.G., Keeling D.M., Laffan M., Tait R.C., Makris M. (2015). Guideline on aspects of cancer-related venous thrombosis. Br. J. Haematol..

[108] Streiff M.B., Holmstrom B., Angelini D., Ashrani A., Bockenstedt P.L., Chesney C., Fanikos J., Fenninger R.B., Fogerty A.E., Gao S. (2018). NCCN Guidelines Insights: Cancer-Associated Venous Thromboembolic Disease, Version 2.2018. J. Natl. Compr. Cancer Netw..

[109] Di Nisio M., Carrier M., Lyman G.H., Khorana A.A. (2014). Prevention of venous thromboembolism in hospitalized medical cancer patients: Guidance from the SSC of the ISTH. J. Thromb. Haemost..

[110] Farge D., Debourdeau P., Beckers M., Baglin C., Bauersachs R.M., Brenner B., Brilhante D., Falanga A., Gerotzafias G.T., Haim N. (2013). International clinical practice guidelines for the treatment and prophylaxis of venous thromboembolism in patients with cancer. J. Thromb. Haemost..

[111] Farge D., Frere C., Connors J.M., Ay C., Khorana A.A., Munoz A., Brenner B., Kakkar A., Rafii H., Solymoss S. (2019). 2019 international clinical practice guidelines for the treatment and prophylaxis of venous thromboembolism in patients with cancer. Lancet. Oncol..

[112] Annibali O., Napolitano M., Avvisati G., Siragusa S. (2018). Incidence of venous thromboembolism and use of anticoagulation in hematological malignancies: Critical review of the literature. Crit. Rev. Oncol. Hematol..

[113] Connors J.M. (2017). Thrombophilia Testing and Venous Thrombosis. N. Engl. J. Med..

[114] Elsebaie M.A.T., van Es N., Langston A., Buller H.R., Gaddh M. (2019). Direct oral anticoagulants in patients with venous thromboembolism and thrombophilia: A systematic review and meta-analysis. J. Thromb. Haemost..

[115] Garber J.E., Halabi S., Tolaney S.M., Kaplan E., Archer L., Atkins J.N., Edge S., Shapiro C.L., Dressler L., Paskett E.D. (2010). Factor V Leiden mutation and thromboembolism risk in women receiving adjuvant tamoxifen for breast cancer. J. Natl. Cancer Inst..

[116] Skeith L. (2018). Anticoagulating patients with high-risk acquired thrombophilias. Hematol. Am. Soc. Hematol. Educ. Program Book.

[117] Munoz-Linares C., Ojeda E., Fores R., Pastrana M., Cabero M., Morillo D., Bautista G., Banos I., Monteserin C., Bravo P. (2014). Paroxysmal nocturnal hemoglobinuria: A single Spanish center's experience over the last 40 yr. Eur. J. Haematol..

[118] Hall C., Richards S., Hillmen P. (2003). Primary prophylaxis with warfarin prevents thrombosis in paroxysmal nocturnal hemoglobinuria (PNH). Blood.

[119] Schubert J.R., Röth A., Bettelheim P., Stüssi G., Höchsmann B., Panse J., Brümmendorf T.H., Schrezenmeier H. Paroxysmale nächtliche Hämoglobinurie (PNH). https://www.onkopedia.com/de/onkopedia/guidelines/paroxysmale-naechtliche-haemoglobinurie-pnh/@@guideline/html/index.html.

[120] Agnelli G., Bergqvist D., Cohen A.T., Gallus A.S., Gent M., investigators P. (2005). Randomized clinical trial of postoperative fondaparinux versus perioperative dalteparin for prevention of venous thromboembolism in high-risk abdominal surgery. Br. J. Surg..

[121] Opatrny L., Warner M.N. (2004). Risk of thrombosis in patients with malignancy and heparin-induced thrombocytopenia. Am. J. Hematol..

[122] Wu W., Wang M., Zhou W., Wang Y. (2020). Heparin-induced thrombocytopenia with hematoma necrosis and persistent high fever after gastric cancer surgery: A case report. Asian J. Surg..

[123] Kreher S., Ochsenreither S., Trappe R.U., Pabinger I., Bergmann F., Petrides P.E., Koschmieder S., Matzdorff A., Tiede A., Griesshammer M. (2014). Prophylaxis and management of venous thromboembolism in patients with myeloproliferative neoplasms: Consensus statement of the Haemostasis Working Party of the German Society of Hematology and Oncology (DGHO), the Austrian Society of Hematology and Oncology (ÖGHO) and Society of Thrombosis and Haemostasis Research (GTH e.V.). Ann. Hematol..

[124] Barbui T., Ghirardi A., Masciulli A., Carobbio A., Palandri F., Vianelli N., De Stefano V., Betti S., Di Veroli A., Iurlo A. (2019). Second cancer in Philadelphia negative myeloproliferative neoplasms (MPN-K). A nested case-control study. Leukemia.

[125] Mustafa S., Stein P.D., Patel K.C., Otten T.R., Holmes R., Silbergleit A. (2003). Upper extremity deep venous thrombosis. Chest.

[126] Flinterman L.E., van Hylckama Vlieg A., Rosendaal F.R., Doggen C.J. (2010). Venous thrombosis of the upper extremity: Effect of blood group and coagulation factor levels on risk. Br. J. Haematol..

[127] Saber W., Moua T., Williams E.C., Verso M., Agnelli G., Couban S., Young A., De Cicco M., Biffi R., van Rooden C.J. (2011). Risk factors for catheter-related thrombosis (CRT) in cancer patients: A patient-level data (IPD) meta-analysis of clinical trials and prospective studies. J. Thromb. Haemost..

[128] Evans R.S., Sharp J.H., Linford L.H., Lloyd J.F., Tripp J.S., Jones J.P., Woller S.C., Stevens S.M., Elliott C.G., Weaver L.K. (2010). Risk of symptomatic DVT associated with peripherally inserted central catheters. Chest.

[129] Bonizzoli M., Batacchi S., Cianchi G., Zagli G., Lapi F., Tucci V., Martini G., Di Valvasone S., Peris A. (2011). Peripherally inserted central venous catheters and central venous catheters related thrombosis in post-critical patients. Intensive Care Med..

[130] Ramot Y., Nyska A. (2007). Drug-induced thrombosis–experimental, clinical, and mechanistic considerations. Toxicol. Pathol..

[131] Ramot Y., Nyska A., Spectre G. (2013). Drug-induced thrombosis: An update. Drug Saf..

[132] Oppelt P., Betbadal A., Nayak L. (2015). Approach to chemotherapy-associated thrombosis. Vasc. Med. Lond. Engl..

[133] Bern M.M., Lokich J.J., Wallach S.R., Bothe A., Benotti P.N., Arkin C.F., Greco F.A., Huberman M., Moore C. (1990). Very low doses of warfarin can prevent thrombosis in central venous catheters. A randomized prospective trial. Ann. Intern. Med..

[134] Monreal M., Alastrue A., Rull M., Mira X., Muxart J., Rosell R., Abad A. (1996). Upper extremity deep venous thrombosis in cancer patients with venous access devices–prophylaxis with a low molecular weight heparin (Fragmin). Thromb. Haemost..

[135] Beckers M.M., Ruven H.J., Seldenrijk C.A., Prins M.H., Biesma D.H. (2010). Risk of thrombosis and infections of central venous catheters and totally implanted access ports in patients treated for cancer. Thromb. Res..

[136] Debourdeau P., Espié M., Chevret S., Gligorov J., Elias A., Dupré P.F., Desseaux K., Kalidi I., Villiers S., Giachetti S. (2017). Incidence, risk factors, and outcomes of central venous catheter-related thromboembolism in breast cancer patients: The CAVECCAS study. Cancer Med..

[137] Lavau-Denes S., Lacroix P., Maubon A., Preux P.M., Genet D., Vénat-Bouvet L., Labourey J.L., Martin J., Slaouti P., Tubiana-Mathieu N. (2013). Prophylaxis of catheter-related deep vein thrombosis in cancer patients with low-dose warfarin, low molecular weight heparin, or control: A randomized, controlled, phase III study. Cancer Chemother. Pharmacol..

[138] Karthaus M., Kretzschmar A., Kröning H., Biakhov M., Irwin D., Marschner N., Slabber C., Fountzilas G., Garin A., Abecasis N.G. (2006). Dalteparin for prevention of catheter-related complications in cancer patients with central venous catheters: Final results of a double-blind, placebo-controlled phase III trial. Ann. Oncol. Off. J. Eur. Soc. Med. Oncol..

[139] Gaitini D., Beck-Razi N., Haim N., Brenner B. (2006). Prevalence of upper extremity deep venous thrombosis diagnosed by color Doppler duplex sonography in cancer patients with central venous catheters. J. Ultrasound Med. Off. J. Am. Inst. Ultrasound Med..

[140] Akl E.A., Karmath G., Yosuico V., Kim S.Y., Barba M., Sperati F., Cook D., Schünemann H.J. (2007). Anticoagulation for thrombosis prophylaxis in cancer patients with central venous catheters. Cochrane Database Syst. Rev..

[141] D'Ambrosio L., Aglietta M., Grignani G. (2014). Anticoagulation for central venous catheters in patients with cancer. N. Engl. J Med..

[142] Heaton D.C., Han D.Y., Inder A. (2002). Minidose (1 mg) warfarin as prophylaxis for central vein catheter thrombosis. Intern. Med. J..

[143] Boraks P., Seale J., Price J., Bass G., Ethell M., Keeling D., Mahendra P., Baglin T., Marcus R. (1998). Prevention of central venous catheter associated thrombosis using minidose warfarin in patients with haematological malignancies. Br. J. Haematol..

[144] Kirkpatrick A., Rathbun S., Whitsett T., Raskob G. (2007). Prevention of central venous catheter-associated thrombosis: A meta-analysis. Am. J. Med..

[145] Rawson K.M., Newburn-Cook C.V. (2007). The use of low-dose warfarin as prophylaxis for central venous catheter thrombosis in patients with cancer: A meta-analysis. Oncol. Nurs. Forum.

[146] Akl E.A., Ramly E.P., Kahale L.A., Yosuico V.E., Barba M., Sperati F., Cook D., Schünemann H. (2014). Anticoagulation for people with cancer and central venous catheters. Cochrane Database Syst. Rev..

[147] Kahale L.A., Tsolakian I.G., Hakoum M.B., Matar C.F., Barba M., Yosuico V.E., Terrenato I., Sperati F., Schünemann H., Akl E.A. (2018). Anticoagulation for people with cancer and central venous catheters. Cochrane Database Syst. Rev..

[148] Mismetti P., Mille D., Laporte S., Charlet V., Buchmuller-Cordier A., Jacquin J.P., Fournel P., Dutrey-Dupagne C., Decousus H., CIP Study Group (2003). Low-molecular-weight heparin (nadroparin) and very low doses of warfarin in the prevention of upper extremity thrombosis in cancer patients with indwelling long-term central venous catheters: A pilot randomized trial. Haematologica.

[149] Couban S., Goodyear M., Burnell M., Dolan S., Wasi P., Barnes D., Macleod D., Burton E., Andreou P., Anderson D.R. (2005). Randomized placebo-controlled study of low-dose warfarin for the prevention of central venous catheter-associated thrombosis in patients with cancer. J. Clin. Oncol. Off. J. Am. Soc. Clin. Oncol..

[150] Ruud E., Holmstrom H., De Lange C., Hogstad E.M., Wesenberg F. (2006). Low-dose warfarin for the prevention of central line-associated thromboses in children with malignancies—A randomized, controlled study. Acta Paediatr..

[151] Verso M., Agnelli G., Kamphuisen P.W., Ageno W., Bazzan M., Lazzaro A., Paoletti F., Paciaroni M., Mosca S., Bertoglio S. (2008). Risk factors for upper limb deep vein thrombosis associated with the use of central vein catheter in cancer patients. Intern. Emerg. Med..

[152] De Cicco M., Matovic M., Balestreri L., Steffan A., Pacenzia R., Malafronte M., Fantin D., Bertuzzi C.A., Fabiani F., Morassut S. (2009). Early and short-term acenocumarine or dalteparin for the prevention of central vein catheter-related thrombosis in cancer patients: A randomized controlled study based on serial venographies. Ann. Oncol. Off. J. Eur. Soc. Med. Oncol..

[153] Young A.M., Billingham L.J., Begum G., Kerr D.J., Hughes A.I., Rea D.W., Shepherd S., Stanley A., Sweeney A., Wilde J. (2009). Warfarin thromboprophylaxis in cancer patients with central venous catheters (WARP): An open-label randomised trial. Lancet.

[154] Niers T.M., Di Nisio M., Klerk C.P., Baarslag H.J., Buller H.R., Biemond B.J. (2007). Prevention of catheter-related venous thrombosis with nadroparin in patients receiving chemotherapy for hematologic malignancies: A randomized, placebo-controlled study. J. Thromb. Haemost..

[155] Debourdeau P., Farge D., Beckers M., Baglin C., Bauersachs R.M., Brenner B., Brilhante D., Falanga A., Gerotzafias G.T., Haim N. (2013). International clinical practice guidelines for the treatment and prophylaxis of thrombosis associated with central venous catheters in patients with cancer. J. Thromb. Haemost..

[156] Zwicker J.I., Connolly G., Carrier M., Kamphuisen P.W., Lee A.Y. (2014). Catheter-associated deep vein thrombosis of the upper extremity in cancer patients: Guidance from the SSC of the ISTH. J. Thromb. Haemos..

[157] Blom J.W., Vanderschoot J.P., Oostindiër M.J., Osanto S., van der Meer F.J., Rosendaal F.R. (2006). Incidence of venous thrombosis in a large cohort of 66,329 cancer patients: Results of a record linkage study. J. Thromb. Haemos..

[158] Falanga A., Marchetti M., Russo L. (2012). Venous thromboembolism in the hematologic malignancies. Curr. Opin. Oncol..

[159] Kristinsson S.Y., Pfeiffer R.M., Björkholm M., Goldin L.R., Schulman S., Blimark C., Mellqvist U.H., Wahlin A., Turesson I., Landgren O. (2010). Arterial and venous thrombosis in monoclonal gammopathy of undetermined significance and multiple myeloma: A population-based study. Blood.

[160] Kristinsson S.Y., Tang M., Pfeiffer R.M., Björkholm M., Goldin L.R., Blimark C., Mellqvist U.H., Wahlin A., Turesson I., Landgren O. (2012). Monoclonal gammopathy of undetermined significance and risk of infections: A population-based study. Haematologica.

[161] Zangari M., Saghafifar F., Mehta P., Barlogie B., Fink L., Tricot G. (2003). The blood coagulation mechanism in multiple myeloma. Semin. Thromb. Hemost..

[162] Elice F., Fink L., Tricot G., Barlogie B., Zangari M. (2006). Acquired resistance to activated protein C (aAPCR) in multiple myeloma is a transitory abnormality associated with an increased risk of venous thromboembolism. Br. J. Haematol..

[163] Rajkumar S.V., Blood E., Vesole D., Fonseca R., Greipp P.R. (2006). Phase III clinical trial of thalidomide plus dexamethasone compared with dexamethasone alone in newly diagnosed multiple myeloma: A clinical trial coordinated by the Eastern Cooperative Oncology Group. J. Clin. Oncol. Off. J. Am. Soc. Clin. Oncol..

[164] Zangari M., Siegel E., Barlogie B., Anaissie E., Saghafifar F., Fassas A., Morris C., Fink L., Tricot G. (2002). Thrombogenic activity of doxorubicin in myeloma patients receiving thalidomide: Implications for therapy. Blood.

[165] Rajkumar S.V., Jacobus S., Callander N.S., Fonseca R., Vesole D.H., Williams M.E., Abonour R., Siegel D.S., Katz M., Greipp P.R. (2010). Lenalidomide plus high-dose dexamethasone versus lenalidomide plus low-dose dexamethasone as initial therapy for newly diagnosed multiple myeloma: An open-label randomised controlled trial. Lancet Oncol..

[166] Baz R., Walker E., Karam M.A., Choueiri T.K., Jawde R.A., Bruening K., Reed J., Faiman B., Ellis Y., Brand C. (2006). Lenalidomide and pegylated liposomal doxorubicin-based chemotherapy for relapsed or refractory multiple myeloma: Safety and efficacy. Ann. Oncol. Off. J. Eur. Soc. Med. Oncol..

[167] Morgan G.J., Schey S.A., Wu P., Srikanth M., Phekoo K.J., Jenner M., Davies F.E. (2007). Lenalidomide (Revlimid), in combination with cyclophosphamide and dexamethasone (RCD), is an effective and tolerated regimen for myeloma patients. Br. J. Haematol..

[168] Palumbo A., Rajkumar S.V., Dimopoulos M.A., Richardson P.G., San Miguel J., Barlogie B., Harousseau J., Zonder J.A., Cavo M., Zangari M. (2008). Prevention of thalidomide- and lenalidomide-associated thrombosis in myeloma. Leukemia.

[169] Li A., Wu Q., Luo S., Warnick G.S., Zakai N.A., Libby E.N., Gage B.F., Garcia D.A., Lyman G.H., Sanfilippo K.M. (2019). Derivation and Validation of a Risk Assessment Model for Immunomodulatory Drug-Associated Thrombosis Among Patients With Multiple Myeloma. J. Natl. Compr. Cancer Netw..

[170] Sanfilippo K.M., Luo S., Wang T.F., Fiala M., Schoen M., Wildes T.M., Mikhael J., Kuderer N.M., Calverley D.C., Keller J. (2019). Predicting venous thromboembolism in multiple myeloma: Development and validation of the IMPEDE VTE score. Am. J. Hematol..

[171] Carrier M., Le Gal G., Tay J., Wu C., Lee A.Y. (2011). Rates of venous thromboembolism in multiple myeloma patients undergoing immunomodulatory therapy with thalidomide or lenalidomide: A systematic review and meta-analysis. J. Thromb. Haemost..

[172] Palumbo A., Cavo M., Bringhen S., Zamagni E., Romano A., Patriarca F., Rossi D., Gentilini F., Crippa C., Galli M. (2011). Aspirin, warfarin, or enoxaparin thromboprophylaxis in patients with multiple myeloma treated with thalidomide: A phase III, open-label, randomized trial. J. Clin. Oncol. Off. J. Am. Soc. Clin. Oncol..

[173] Larocca A., Cavallo F., Bringhen S., Di Raimondo F., Falanga A., Evangelista A., Cavalli M., Stanevsky A., Corradini P., Pezzatti S. (2012). Aspirin or enoxaparin thromboprophylaxis for patients with newly diagnosed multiple myeloma treated with lenalidomide. Blood.

[174] Wang T.F., Zwicker J.I., Ay C., Pabinger I., Falanga A., Antic D., Noble S., Khorana A.A., Carrier M., Meyer G. (2019). The use of direct oral anticoagulants for primary thromboprophylaxis in ambulatory cancer patients: Guidance from the SSC of the ISTH. J. Thromb. Haemost..

[175] Storrar N.P.F., Mathur A., Johnson P.R.E., Roddie P.H. (2019). Safety and efficacy of apixaban for routine thromboprophylaxis in myeloma patients treated with thalidomide- and lenalidomide-containing regimens. Br. J. Haematol..

