# Supplementary material for: Primary Thromboprophylaxis in Patients with Malignancies: Daily Practice Recommendations by the Hemostasis Working Party of the German Society of Hematology and Medical Oncology (DGHO), the Society of Thrombosis and Hemostasis Research (GTH), and the Austrian Society of Hematology and Oncology (ÖGHO)
Source: Cancers (Basel). 2021 Jun 10;13(12):2905. doi: 10.3390/cancers13122905 (PMC8230401; doi:10.3390/cancers13122905)
Supplement: Supplementary file 1 [file cancers-13-02905-s001.zip › cancers-1231741-supplementary.pdf]

cancers

MDPI

Table 1. Combined Presentation of All Recommendations Sorted by Chapter.

**3.1.**	**Studies in unselected cancer patient cohorts**	
	recommendation:	• Based on recommendations for hospitalized patients due to medical illness in general, we recommend prophylactic anticoagulation for cancer patients in an in-patient setting, except for fit/mobile cancer patients admitted due to non-surgical procedures. In these cases, recommendations for outpatients should be followed (see below).<br>• Due to the wide variability of VTE risk in ambulatory cancer patients, we do not recommend primary pharmacological prophylaxis in general. This topic will be discussed in the following paragraphs. Refer also to figure 1.
**3.2.**	**Studies in selected cancer patient cohorts:**	
	recommendation:	• The available evidence is in favor of primary pharmacological prevention (LMWH either prophylactic or half-therapeutic dose in certain cancer types such as pancreatic cancer, or apixaban 2 × 2.5mg or rivaroxaban 1 × 10mg) in ambulatory cancer patients undergoing systemic anti-cancer therapy with a Khorana score (KS) of > 2 or, although less evidence-based, those identified with other risk scores or with other high risk factors for VTE. However, given the limited available data, the panel agreed that any such recommendation must be made on an individual basis, and it should take into account the patient´s preference.
**3.3.**	**Drug Interactions in patients with malignancies receiving prophylactic anticoagulation**	
	recommendation:	• If prophylactic anticoagulation is indicated, the choice of optimal oral drug should be considered on an individual basis taking into account drug interactions and the bioavailability (e.g. patients after abdominal surgery). In uncertain cases, LMWH should be preferred to DOACs.
**4.1.**	**Peri-interventional thromboprophylaxis in patients with cancer undergoing elective invasive procedures**	
	recommendation:	• Prophylactic anticoagulation in cancer patients undergoing major abdominal or pelvic surgery should be extended to 4-5 weeks after surgery.<br>• Thromboprophylaxis should be performed with LMWH, UFH (second choice), or fondaparinux. The exact peri-operative timing of these drugs is described in the respective SmPC.<br>• Mechanical prophylaxis (compression stockings or intermittent pneumatic compression) may be performed in patients with contraindications for anticoagulation.
**4.2.**	**Thromboprophylaxis in patients with cancer and renal insufficiency**	
	recommendation:	• In patients with renal dysfunction, the risk of bleeding during thromboprophylaxis is increased. However, no firm clinical evidence exists on how to adjust the dose of thromboprophylactic agents. Therefore, we recommend a very thorough assessment of the necessity for and the dose of pharmacologic thromboprophylaxis in patients with reduced eGFR, particularly those with an eGFR below 30 ml/min. The risks of bleeding have to be weighed against the risk of thromboembolism.<br>• Anti-Xa plasma level monitoring during LMWH thromboprophylaxis is

		helpful in patients with stage 4 to 5 chronic kidney disease, with optimal levels (3-4h after injection) being 0.1 to 0.3 IU/mL for prophylactic dosing[79]. However, clinical judgement currently remains the main factor in deciding on the dose of these agents.
**4.3.**	**Thromboprophylaxis in patients with cancer and liver disease**	
	recommendation:	• There are no evidence-based recommendations on prophylaxis of cancer-associated VTE in patients with severe liver disease. We recommend individualized treatment regimens after shared decision-making with patients.<br>• Prophylactic dose anticoagulation may usually be offered since the bleeding risk is low. Careful clinical and laboratory monitoring is suggested, especially in patients with prolonged prothrombin time.<br>• In patients with liver disease and a history of severe bleeding, e.g. from esophageal varices, it might be prudent not to use pharmacological thromboprophylaxis.<br>• Make sure the patient has been informed about clinically relevant bleeding symptoms and about signs of thromboembolism. He/She should know whom to contact when developing such symptoms and where to go after office hours or on the weekend.
**4.4.**	**Thromboprophylaxis in patients with cancer and elevated bleeding risk**	
	recommendation:	• Tumor-site specific risk: an individual assessment of bleeding risk has to be carried out, especially in patients with glioblastomas, lymphomas, gastrointestinal cancers, lung cancers and tumors of the genitourinary tract.<br>• Individual/situational risk: laboratory tests reflecting organ function and platelet count as well as age, bleeding history have to be evaluated carefully. In the context of interventional procedures, management and scoring of bleeding risk is recommended for individual adaptation of thromboprophylaxis.<br>• Drug-specific risk factors: bleeding risk has to be assessed and monitored according to the anti-cancer medication and other co-medication.
**4.5.**	**Thromboprophylaxis in patients with cancer and thrombocytopenia**	
	recommendation:	In thrombocytopenic patients:<br>• we recommend against thromboprophylaxis in cancer patients, if platelet counts are < 25 G/l.<br>• pharmacologic thromboprophylaxis with LMWH may be considered, if platelet counts are between 25 and 50 G/l.<br>• we recommend against mechanical prophylaxis (pneumatic intermittent compression or compression stockings) in patients with platelet counts < 50 G/l.

**4.6.**	**Thromboprophylaxis in patients with cancer and thrombophilia**	
	recommendation:	• Patients with malignancies and co-existing thrombophilia require special attention and potentially alternative prophylactic anticoagulation. However, prospective data on prophylactic anticoagulation are lacking. We thus recommend to adhere to guidelines for non-cancer patients with thrombophilia.<br>• Breast cancer patients receiving tamoxifen treatment have an increased VTE risk if they have a heterozygous factor V Leiden mutation, and we recommend genetic testing if this information is likely to change clinical management. There are no general guidelines on thromboprophylaxis in these patients.<br>• We recommend thromboprophylaxis in patients with cancer and homozygous or combined (compound) factor V and/or prothrombin mutations or antithrombin, protein C, or protein S deficiency, if they have a Khorona-Score ≥ 2 or other thrombogenic risk factors.<br>• We recommend routine prophylactic anticoagulation with LMWH in high-risk situations for patients without previous thromboembolism who have a heterozygous factor V Leiden mutation or a heterozygous prothrombin mutation G20210A. Whether DOACs are equally efficacious, has yet to be determined, but they have been used in cancer patients, and the studies did not exclude patients with thrombophilia.<br>• Patients with thrombotic APS and cancer require long-term therapeutic anticoagulation with vitamin K antagonists or adjusted doses of LMWH +/- aspirin. DOACs must be avoided in high-risk APS (i.e. triple-positive APS) and should also be avoided in low-risk APS.<br>• We recommend thromboprophylaxis in patients with PNH and cancer. However, it has to be weighed against the risk of bleeding and the potential of other drugs (e.g. eculizumab) to prevent thromboembolism.<br>• Patients with certain Bcr-Abl negative MPNs (all PV, int-/high-risk ET) should receive acetylsalicylic acid (ASA) and potentially additional primary VTE prophylaxis (individual decision for LMWH or DOAC).
**4.7.**	**Thromboprophylaxis in patients with cancer and indwelling catheters**	
	recommendation:	• It is not generally recommended to administer prophylactic anticoagulation in ambulatory cancer patients solely because of implanted central venous catheters.<br>• The role of DOACs in this setting remains unclear.
**4.8.**	**Thromboprophylaxis in patients with multiple myeloma**	

	recommenda-tion:	• VTE risk assessment is recommended in patients with MM at diagnosis and during the course of the disease.<br>• The SAVED score (for patients treated with IMiDs) and the IMPEDE-VTE score (independent of IMiD therapy) are recommended as validated VTE risk assessment tools and classify into low and high VTE risk. In low-risk patients, ASA or no intervention is recommended, whereas prophylaxis with LMWH is recommended in high-risk patients.<br>• The use of DOACs in high-risk patients is being examined in current studies
